# Distributions, interactions, and dynamics of prokaryotes and phages in a hybrid biological wastewater treatment system

**DOI:** 10.1186/s40168-024-01853-6

**Published:** 2024-07-22

**Authors:** Dou Wang, Lei Liu, Xiaoqing Xu, Chunxiao Wang, Yulin Wang, Yu Deng, Tong Zhang

**Affiliations:** 1 Environmental Microbiome Engineering and Biotechnology Laboratory, Center for Environmental Engineering Research, Department of Civil Engineering, The University of Hong Kong, Hong Kong SAR, China; 2 School of Public Health, The University of Hong Kong, Hong Kong SAR, China; 3 Macau Institute for Applied Research in Medicine and Health, Macau University of Science and Technology, Macau SAR, China

**Keywords:** Prokaryote, Phage, Host-phage interactions, Hybrid system, Multi-omics, Hi-C sequencing

## Abstract

**Background:**

Understanding the interactions and dynamics of microbiotas within biological wastewater treatment systems is essential for ensuring their stability and long-term sustainability. In this study, we developed a systematic framework employing multi-omics and Hi-C sequencing to extensively investigate prokaryotic and phage communities within a hybrid biofilm and activated sludge system.

**Results:**

We uncovered distinct distribution patterns, metabolic capabilities, and activities of functional prokaryotes through the analysis of 454 reconstructed prokaryotic genomes. Additionally, we reconstructed a phage catalog comprising 18,645 viral operational taxonomic units (vOTUs) with high length and contiguity using hybrid assembly, and a distinct distribution of phages was depicted between activated sludge (AS) and biofilm. Importantly, 1340 host-phage pairs were established using Hi-C and conventional in silico methods, unveiling the host-determined phage prevalence. The majority of predicted hosts were found to be involved in various crucial metabolic processes, highlighting the potential vital roles of phages in influencing substance metabolism within this system. Moreover, auxiliary metabolic genes (AMGs) related to various categories (e.g., carbohydrate degradation, sulfur metabolism, transporter) were predicted. Subsequent activity analysis emphasized their potential ability to mediate host metabolism during infection. We also profiled the temporal dynamics of phages and their associated hosts using 13-month time-series metagenomic data, further demonstrating their tight interactions. Notably, we observed lineage-specific infection patterns, such as potentially host abundance- or phage/host ratio-driven phage population changes.

**Conclusions:**

The insights gained from this research contribute to the growing body of knowledge surrounding interactions and dynamics of host-phage and pave the way for further exploration and potential applications in the field of microbial ecology.

Video Abstract

**Supplementary Information:**

The online version contains supplementary material available at 10.1186/s40168-024-01853-6.

## Background

The hybrid moving bed biofilm reactor (HMBBR), where the suspended activated sludge (AS) and attached biomass (biofilm) on carriers co-exist, represents an optimized wastewater treatment system that combines the merits of both AS and biofilm processes, resulting in improved overall efficiency and stability of the treatment process [1]. Within the HMBBR system, AS contributes to the removal of organic pollutants and nutrients from wastewater through microbial flocs or granules involved in organic matter degradation, nitrification, denitrification, and polyphosphate accumulation processes [2–4]. Compared with the AS process, the attached biofilms are effective at accumulating slow-growing nitrifiers and provide anoxic/anaerobic conditions that are favorable for the habitation of anaerobic microorganisms and denitrifiers [5, 6]. By integrating the features of these two processes, the hybrid system possesses the potential to foster a more complex microbial community, owing to the presence of distinct microbial assemblies in both environments. Given their distinct microbial assemblies, gaining insight into the assembly and ecology of phage communities in both AS and biofilm is of great interest.

Recent studies have shed light on the huge diversity and ecology of phages in the AS treatment process [7–10]. In our recent study, approximately 50,000 phage sequences were reconstructed from AS samples collected from six wastewater treatment plants (WWTPs) in Hong Kong [7]. This research not only uncovered the tremendous phage diversity but also demonstrated that they could potentially influence nutrient removal and the carbon cycle within the biological wastewater treatment system by regulating microbial community and auxiliary metabolism. Fan et al. investigated the diversity and biogeography of phages in global AS systems, which further confirmed the presence of the highly diverse phage taxonomic groups and emphasized their potential ecological roles in AS systems [10]. Nonetheless, the assembly of phages in the biological biofilm system, a more biomass-dense ecosystem, remains unexplored. In comparison to the AS system, biofilms are denser and more heterogeneous, and these characteristics suggest the possibility of distinct phage distribution and ecology within this system [11]. By utilizing a biofilm simulation framework, Simmons et al. demonstrated that factors like nutrient availability, the probability of infection per host encounter, and phage diffusion ability can significantly influence the interactions between phages and hosts within biofilm [12]. Moreover, Hwang et al. have highlighted that the prolonged contact of diverse microbes, as well as limited host and viral dispersal due to biofilm characteristics, can affect host-phage interactions [11]. For instance, these factors may promote simultaneous infection involving multiple hosts. Hence, exploring the phage infection pattern within the biological biofilm system is crucial for understanding their ecological roles within this system.

To fully understand the potential ecological roles of phages in biological wastewater treatment systems, it is essential to reconstruct phage genomes and establish prokaryote-phage linkages from this complex system. At present, the virus-like particle (VLP) enrichment approach has enhanced the recovery of phages from complex environments [7, 13–15]. This approach tackles the difficulties in recovering phages from environmental samples that arise due to the highly complex nature of samples and the presence of numerous host cells. Moreover, by incorporating advanced sequencing strategies, like long-read sequencing, the reconstruction of phage genomes with high completeness and length becomes possible [16–18]. In addition, the advancement of approaches for host-phage pair determination, such as Hi-C, enables a robust and high-throughput approach to capture the proximity signal of phage and host DNA during infection [19]. Phage DNA can be physically connected with the host DNA through in vivo crosslinking, which further involves the cleavage of cross-linked DNA and re-ligation to identify which fragments of DNA were in close spatial proximity within the cell, thereby confirming their internal relationship [20–22]. Currently, this approach has been successfully used in complex environmental systems for host-phage pair determination [11, 20].

In this study, we aim to get a comprehensive understanding of the distributions and interactions of the prokaryotic and phage communities within a hybrid system. Illumina short-read and nanopore long-read sequencing were combined to establish the genome catalogs of prokaryotes and phages. The newly reconstructed 454 prokaryotic genomes and 18,645 viral operational taxonomic units (vOTUs) demonstrated the distinct distribution and diversity of prokaryotic and phage communities between AS and biofilm. Furthermore, the extensive host-phage interactions revealed by integrating in silico prediction methods and Hi-C sequencing shed light on their close connections and potential roles in regulating the microbial community and metabolism. Notably, a time-series metagenome dataset spanning a 13-month period was utilized to uncover the phage-host infection dynamics and the potential effects of phage infection on system stability. Overall, this study offers a comprehensive understanding of the prokaryote and phage distribution, diversity, and interaction patterns, including coexistence or infection dynamics, within the full-scale hybrid biofilm and activated sludge system.

## Methods

### Sample collection

In the present study, AS and carrier biofilm samples were simultaneously collected from an HMBBR at Stanley Sewage Treatment Works in Hong Kong on June 29, 2021. The details of this treatment work are provided in Additional file 1. AS samples were collected from four tanks (named A, B, C, and D) along the flow path, while carriers were collected from tank B, referred to as TB-c. The treatment work setup and sampling scheme are depicted in Additional file 1: Fig. S1. Immediately following collection, samples were rapidly frozen in liquid nitrogen, delivered to the lab, and preserved at − 80 ℃ for subsequent DNA and RNA extraction and sequencing. Furthermore, water samples were taken at each sampling site and filtered using a 0.45-um membrane for chemical analysis. The detailed chemical parameters of different sampling points are listed in Additional file 2: Table S1.

### Nucleotide extraction and sequencing

For the carrier sample, the attached biofilm was carefully peeled off using a clean brush before the extraction procedure. The total genomic DNA and RNA of AS and carrier biofilm samples were extracted using the DNeasy® PowerSoil® Kit (Qiagen, Hilden, Germany) and RNeasy® PowerSoil® Total RNA Kit (Qiagen, Hilden, Germany), respectively, following the manufacturer’s instructions. The concentration and purity of extracted DNA were assessed using the Qubit assay (Thermo Fisher Scientific) on a Qubit® 2.0 Fluorometer (Invitrogen Life Technologies, NY, USA) and NanoDrop (Thermo Fisher Scientific, USA), respectively. For Illumina sequencing, the NovaSeq PE150 platform was used to generate 150-bp paired-end reads with a 350-bp insert size at Novogene (Beijing, China). For nanopore sequencing, the library was prepared using the ligation sequencing kit (SQK-LSK109) and sequenced using R9.4.1 flow cells (FLO-MIN106) on the GridION sequencer (Oxford Nanopore Technology, UK). For metatranscriptomic sequencing, the Ribo-Zero rRNA removal kit (Illumina, USA) was initially used to eliminate rRNA. The remaining RNAs were fragmented into around 250–300 bp and then reverse-transcribed into double-stranded cDNAs, which were then sequenced to generate paired-end reads, as mentioned above. For metagenomic sequencing, approximately 30–60 Gb of sequencing data were generated for each sample. In the case of metatranscriptomic sequencing, around 10 Gb of data were produced for each sample.

### Metagenomic analysis

Before downstream analysis, metagenomic paired-end reads from AS and biofilm samples were quality-checked using fastp (v0.20.1) [23] with default parameters. To gain an overview of the prokaryotic and eukaryotic composition of each sample, phyloFlash (v3.4.1) [24] was initially employed for the taxonomic assignment of the communities based on the metagenomic short reads, using SILVA SSU Ref database (v.138.1) [25]. Furthermore, two different assembly approaches were utilized to recover representative genomes from these samples, including the iterative haplotype-resolved hierarchical clustering-based hybrid assembly (HCBHA) approach developed by our group [26] and OPERA-MS (v0.8.3) [27]. Subsequently, the metagenome-assembled genomes (MAGs) retrieved from different cycles and methods were dereplicated using dRep (v2.6.2) [28] with an average nucleotide identity (ANI) cutoff of 99%. CheckM (v1.1.3) [29] was used to estimate the quality of the MAGs, and only MAGs with completeness of ≥ 50% and contamination of ≤ 10% were selected for further analyses.

### Phylogenetic analysis, MAG annotation, and metabolic prediction

GTDB-Tk (v2.3.2, reference data version r214) [30, 31] was utilized for the taxonomic classification and phylogeny inference of the newly recovered MAGs. FastTree (v2.1.10) [32] was used to infer the genome tree generated by GTDB-Tk. The resulting tree was then visualized using the Interactive Tree of Life (iTOL) (https://itol.embl.de/) [33]. Prodigal (v2.6.3) [34] was used for open reading frame (ORF) prediction of the recovered MAGs. The genes and metabolic traits of the newly recovered MAGs were annotated using the Kyoto Encyclopedia of Genes and Genomes (KEGG) GhostKOALA [35].

To predict the metabolic potential of these reconstructed MAGs, METABOLIC (v4.0) [36] was employed. In order to estimate the specific community-level metabolic capacity or activity, the number and relative abundance or activity of MAGs in the community capable of performing a specific metabolic function were summed. A MAG with a relative abundance exceeding 0.01% is considered present in a sample. CoverM (v0.4.0) [37] was used to calculate the abundance and expression of individual microbes by mapping the metagenomic and metatranscriptomic reads to the retrieved MAGs, using parameters of 90% read-percent-identity and 80% read-aligned-percent. Before metatranscriptomic analysis, SortMeRNA (v4.0.0) [38] was applied to remove non-coding RNA sequences from the metatranscriptomic data.

### Viral sample collection, enrichment, and processing

For phage catalog establishment, AS and carrier samples were collected simultaneously on June 29, 2021. Approximately 5 L of AS slurry was collected from each tank (A, B, C, and D), and carriers were collected from tank B (TB-c) (Additional file 1: Fig. S1). The detailed filtration and purification process is described in Additional file 1. The DNA of the enriched sample was extracted using the phage DNA isolation kit (Norgen Biotek Corp., Canada) with proteinase K, following the manufacturer’s instructions. The concentration and purity of the extracted DNA were assessed using NanoDrop (Thermo Fisher Scientific, USA). DNA samples were then stored at − 80 ℃ for subsequent analysis. The procedures for Illumina and nanopore sequencing are the same as for the sequencing section above. Approximately 37 Gb of nanopore long-read data and 55 Gb of Illumina short-read data were generated from the five VLP-enriched samples.

For the Hi-C sequencing, the AS and carrier biofilm samples were first crosslinked with a 1% final formaldehyde concentration; the mixture was incubated for 20 min at room temperature with periodic mixing. Formaldehyde was quenched by adding excess glycine and incubated for 15 min at room temperature with periodic mixing. Samples were then recovered by centrifugation, rinsed with PBS, re-centrifuged, and collected samples were stored at − 80 ℃. Subsequently, the fixed samples were delivered to Phase Genomics (Seattle, WA 98109, USA) for subsequent treatment, sequencing, and analysis. The detailed information of Hi-C library preparation is described in Additional file 1.

### Assembly and prediction of viral operational taxonomic units

Three different assembly approaches were used in this study, including short-read assembly, Flye [39] assembly, and OPERA-MS [27] assembly. The detailed assembly processes are described in Additional file 1. Assembled contigs from short-read and two hybrid strategies were subjected to phage sequence prediction. In this study, Virsorter2 (v2.2.2) [40] and DeepVirFinder (v1.0) [41] were used for phage sequence identification. To obtain high-confidence phage sequences, assembled contigs ≥ 5 kb in length were reserved for analysis, and conservative settings for both tools were adopted. With DeepVirFinder, contigs with a score ≥ 0.9 and *p* < 0.05 were identified as putative phages [42]. The prediction results of these two methods were combined to get the putative phage sequences of each assembly. Subsequently, the putative phages predicted from these three assembly approaches were clustered to yield non-redundant representativevOTUs by performing UCLUST-like clustering using the MIUViG recommended parameters of 95% average nucleotide identity over 85% alignment fraction (relative to the shorter sequence) [43]—the codes used here were available at the CheckV website (https://bitbucket.org/berkeleylab/checkv/src/master/).

### vOTU classification, annotation, and auxiliary metabolic gene prediction

The taxonomy classification of retrieved vOTUs was conducted based on gene-sharing network clustering using vConTACT2 (v0.11.3) [44] with the database version “ProkaryoticViralRefSeq211-Merged”. Besides, PhaGCN2 (v2.0) [45] based on the ICTV database was also used for taxonomy classification. DRAM-v, the viral mode embedded by DRAM (v1.2.4) [46], was employed for the annotation of vOTUs and prediction of auxiliary metabolic genes (AMGs). Prior to the DRAM-v workflow, CheckV (v0.9.0) [47] was first used to remove the potential contamination from the host to get the cleaned phage sequences. Putative AMGs with both auxiliary scores below four and accompanying gene descriptions were retained for further manual curation. Based on the DRAM-v annotation results, genes related to nucleotide metabolism, organic nitrogen, glycosyl transferases, and ribosomal proteins were excluded from subsequent activity analysis [48, 49]. To profile the abundance and activity of AMGs, bulk metagenomic and metatranscriptomic reads were mapped to all vOTU genes using RSEM (v1.3.3) [50] to get the genes per million (GPM) and transcripts per million (TPM) values. BACPHLIP [51] and PhaTYP [52] were used to classify the lytic or lysogenic phages. The prediction results from PhaTYP will be given priority. If PhaTYP is unable to provide a prediction, the results from BACPHLIP will be utilized as an alternative.

### Diversity and abundance analysis of vOTUs

To depict the distribution and diversity of phages, metaviromic and bulk metagenomic reads were mapped to the identified phage genomes to calculate the coverage (divided by the phage genome size) using CoverM (v0.4.0) [37] with the mode of “-mean”. A phage in a metavirome and bulk metagenome will be considered present if the phage coverage exceeds 0.75 and 0.1, respectively. The phage coverage values used in this study were further normalized by the dataset volume, defined as coverage per Gb data. The normalized coverage matrix was then used for Shannon and Simpson index calculation and principal coordinate analysis (PCoA).

### Prediction of host-phage associations

To obtain a comprehensive host-phage association network, an integrated approach consisting of homologous sequence match, transfer RNA (tRNA) match, CRISPR spacer match, and Hi-C pair connections was applied. For homologous sequence match, the contigs of recovered MAGs were compared with phage sequences using BLASTn (≥ 90% identity, matches > 500 bp) [53]. For tRNA match, tRNAs in phage genomes were identified using tRNAscan-SE (v2.0.9) [54] with the general tRNA model (option -B) and default parameters. These detected tRNAs were then queried against the recovered MAGs using BLASTn, requiring 100% query coverage and 100% sequence identity [55]. For the CRISPR spacer match, spacers were identified in the recovered MAGs using the CRISPR Recognition Tool (CRT) [56]. The extracted spacers were then searched against phage genomes using BLASTn (BLASTn-short, ≥ 97% identity, ≥ 90% coverage, and ≤ 1 mismatch) [7]. To detect Hi-C linkages, the ProxiMeta platform, developed by Phase Genomics (Seattle, WA 98109, USA), was used for the establishment of host-phage pairs based on Hi-C data. The detailed information of host-phage linkage matching is described in Additional file 1.

### Analysis of host-phage dynamics

A 13-month time-series AS metagenomic dataset was employed to analyze host-phage dynamics. The AS samples were collected monthly from January 2018 to January 2019 and have previously been used for resistome and mobilome analyses in the same system [57]. The coverage of prokaryotes and phages was calculated by mapping metagenomic data to the MAGs and vOTUs using CoverM (v0.4.0) [37], using parameters of 90% read-percent-identity and 80% read-aligned-percent. The coverage values were further normalized by the metagenomic data size. Procrustes analysis [58] was conducted to investigate the relationships between the prokaryotic and phage communities using the generated coverage matrices. This analysis was performed using the OmicShare tools, a free online platform for data analysis (https://www.omicshare.com/tools). Lineage-specific phage/host ratios were computed based on the predicted host-phage pairs at the phylum level.

## Results

### Microbial community structures in AS and biofilm

**Fig. 1:**
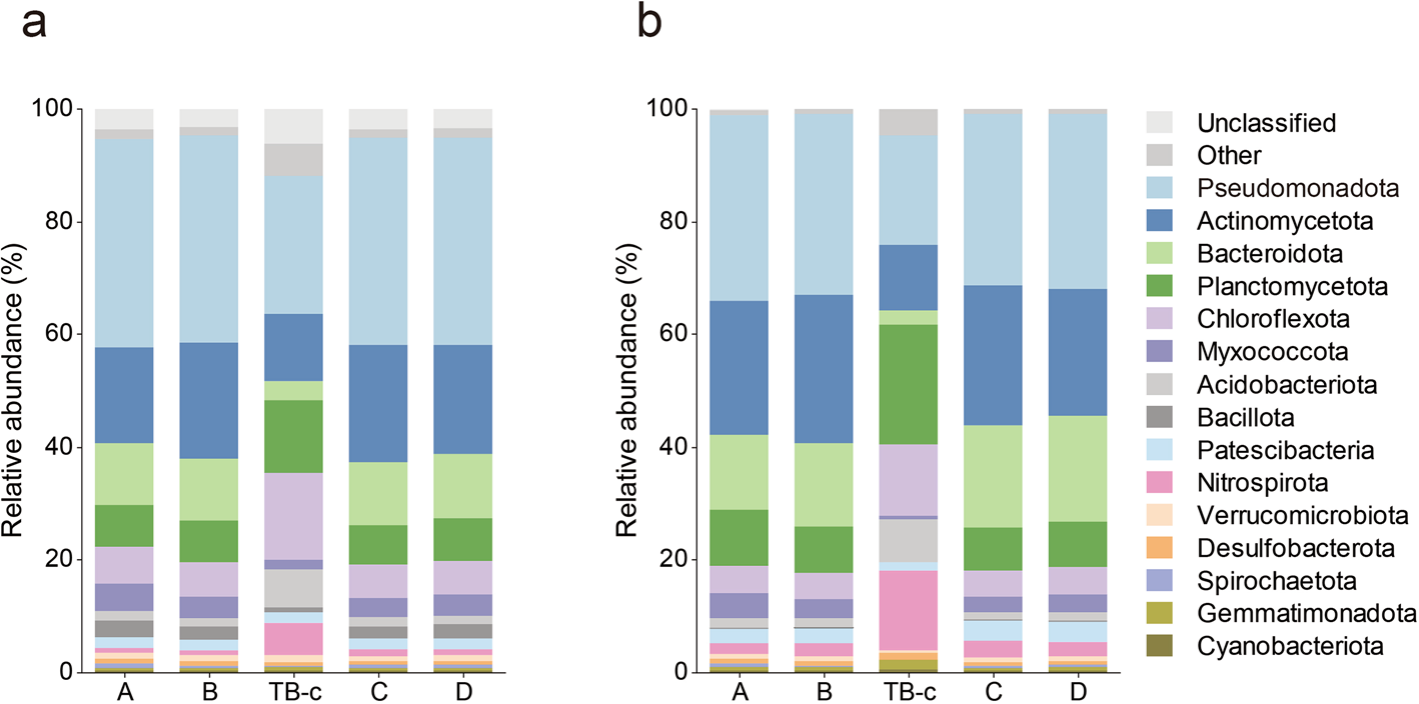
Microbial assembly in AS and carrier biofilm. **a** The relative abundance of the top 15 phyla within the bacterial and archaeal communities, which was derived from the taxonomic assignment of metagenomic 16S rRNA gene reads. **b** The relative abundance of the top 15 phyla within the bacterial and archaeal communities, which was determined by mapping the metagenomic reads to the recovered MAGs

We first aimed to compare the overall microbial community structures and diversities of AS (A, B, C, D) and biofilm (TB-c) samples. The marker gene-based classification results indicated that bacteria dominate all samples, accounting for 84.0–94.6% of the recruited rRNA gene reads, followed by eukaryotic communities (5.3–15.5%) (Additional file 1: Fig. S2). Samples A–D show a similar pattern, and TB-c had proportionally more eukaryotes. Furthermore, distinct patterns of bacterial compositions at the phylum level were revealed between AS and biofilm samples. However, no remarkable distinction was observed among AS samples. The bacterial members belonging to phyla *Pseudomonadota*, *Bacteroidota*, *Actinomycetota*, *Planctomycetota*, and *Chloroflexota* dominated both AS and biofilm samples, accounting for 68.0–81.7% of the total community composition (Fig. 1a). Notably, a higher relative abundance of *Chloroflexota*, *Nitrospinota*, and *Acidobacteriota* was observed in biofilm.

The reconstructed MAGs from multiple assembly cycles, using hybrid assembly, were combined and dereplicated at 99% of ANI, ultimately resulting in a dereplicated set of 454 MAGs (≥ 50% completeness and ≤ 10% contamination) (Additional file 2: Table S2). Approximately 49.8–51.5% of metagenomic reads of AS samples could map to the final bin set, while a lower proportion (31.0%) of biofilm reads could be recruited. The final genome set included 147 high-quality MAGs and 307 medium-quality MAGs (Additional file 1: Fig. S3), classified according to the criteria defined by MIMAG standards [59]. In line with the classification result of miTags, these recovered MAGs spanned 26 known phyla, primarily affiliated with the phyla of *Pseudomonadota* (*n* = 131), *Bacteroidota* (*n* = 57), *Actinomycetota* (*n* = 54), *Planctomycetota* (*n* = 54), and *Chloroflexota* (*n* = 42) (Additional file 1: Fig. S3 and Additional file 2: Table S2). Notably, the GTDB-Tk classification result indicated that 12 MAGs were not able to be assigned to the known family (Additional file 2: Table S2), and approximately 97 MAGs could not find close genomes at the genus level. Only 77 of the 454 recovered MAGs could be annotated at the species level. These findings highlight the high novelty of the recovered genomes.

The microbial community compositions at the phylum level, as interpreted by recovered MAGs (Fig. 1b), were comparable to the profiles illustrated by miTags (Fig. 1a), although a slight variation in the relative abundance of some predominant phyla was observed. The relative abundances of recovered MAGs (Additional file 2: Table S3) were depicted in the phylogenetic tree (Additional file 1: Fig. S3), where remarkable variations in the relative abundance of specific microorganisms were observed. For instance, anaerobic ammonium oxidation (anammox) bacteria were only detected in the carrier biofilm, accounting for about 5% of the entire microbial community. Additionally, two complete ammonia oxidation (comammox) bacteria were identified and dominated the carrier biofilm. Overall, distinct microbial distribution patterns were observed between AS and biofilm.

### Distinct metabolic capacities between AS and biofilm

The differences in the microbial communities can lead to variations in metabolic capacities. As such, we further investigated and compared the metabolic potentials and metabolic activities of the microbial communities involved in the biogeochemical cycling of carbon, nitrogen, and sulfur across the biofilm and AS samples. Among the recovered MAGs from this ecosystem, the majority are capable of organic carbon oxidation (446 MAGs), fermentation (417 MAGs), acetate oxidation (354 MAGs), and sulfur oxidation (243 MAGs) (Additional file 1: Fig. S4). For nitrogen cycling, three recovered MAGs were predicted to be involved in the ammonia oxidation process, including two comammox *Nitrospira* and one ammonia-oxidizing bacteria (AOB). Additionally, 51 MAGs were found to encode genes for nitrite oxidation, with eight of these nitrite oxidizers affiliated with nitrite-oxidizing bacteria (NOB) and two MAGs affiliated with comammox *Nitrospira*. Only one MAG capable of sulfite reduction was recovered in this study.

**Fig. 2:**
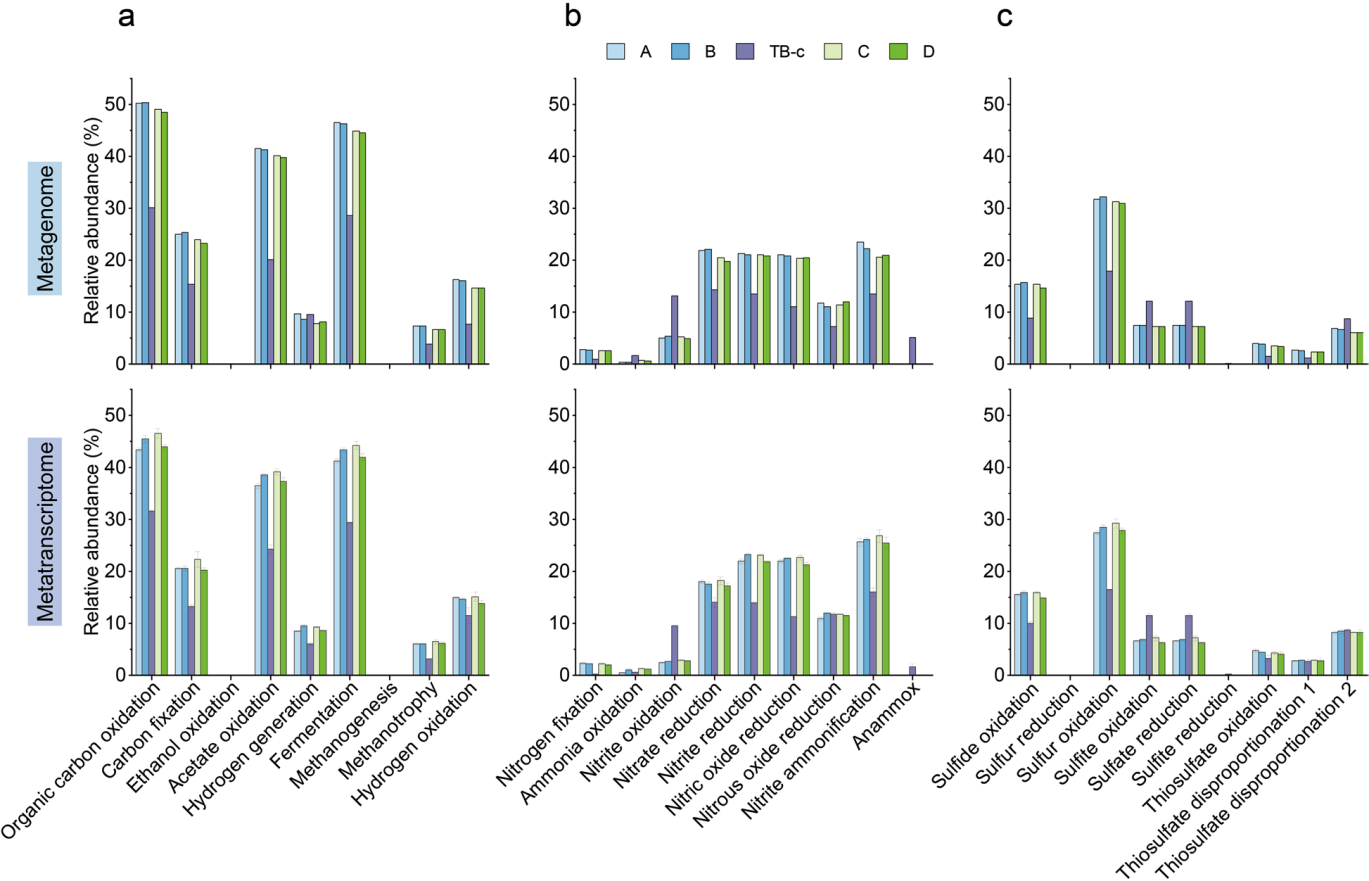
Metabolic profiles of the hybrid communities. The community-level metabolic capacity/activity for carbon (**a**), nitrogen (**b**), and sulfur (**c**) cycling across carrier biofilm and AS samples. The relative abundances of all genomes capable of a specific metabolic function were summed to profile the community-level capacity. The metabolic activities of all genomes capable of a specific metabolic function were summed to profile the community-level activity

The metabolic capacity prediction results indicated that the microbial communities of AS samples encode a greater capacity for all existing metabolic functions involved in carbon cycling, except for hydrogen generation (Fig. 2a). Consistent high metabolic activities of these metabolic functions were also revealed (Fig. 2a). For nitrogen cycling, higher capacities for ammonia oxidation, nitrite oxidation, and anammox reactions were observed in the carrier biofilm community (Fig. 2b). The abundant, newly recovered comammox *Nitrospira* and diverse NOB might contribute to the higher capacity and activity for nitrite oxidation in the biofilm community. In contrast, microbial communities of AS exhibited greater capacities for denitrification, nitrogen fixation, and nitrite ammonification (Fig. 2b). Organisms capable of sulfite oxidation and sulfate reduction were abundant and active in the biofilm community (Fig. 2c). Notably, a higher H_2_S production capacity through thiosulfate disproportionation was observed in biofilm. However, microbes capable of reducing sulfur were absent in this system.

### Diverse and novel phages in this hybrid biofilm and activated sludge system

To create a more comprehensive phage catalog, vOTUs identified from three different assemblers were combined and de-replicated to generate the final set of vOTUs. The detailed information on the vOTUs derived from various approaches is summarized in Additional file 2: Table S4. Following clustering, 18,645 vOTUs were obtained, with a longer average length of 28,038 bp and N50 of 42,298 bp (Additional file 2: Table S4, S5). Approximately 62.0–92.1% of metaviromic reads could be recruited by the final vOTUs set (Additional file 2: Table S4), suggesting that this set could represent the majority of phages enriched from this system.

**Fig. 3:**
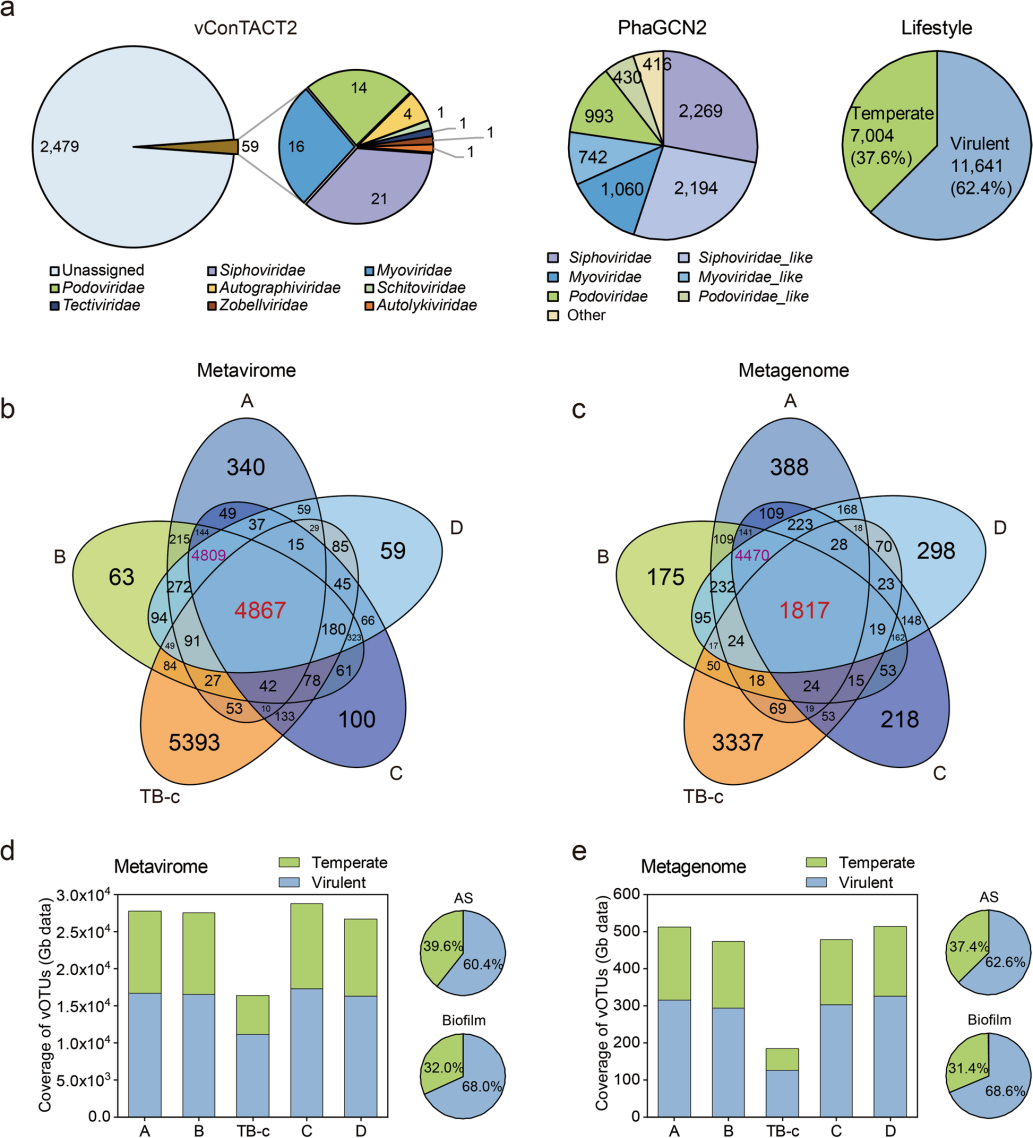
Taxonomic diversity and distribution of vOTUs. **a** Classification and lifestyle prediction of the reconstructed vOTUs. **b** Venn diagram of vOTUs in metavirome. **c** Venn diagram of vOTUs in metagenome. **d** Coverage and proportion of virulent and temperate phages in AS and biofilm metaviromes. **e** Coverage and proportion of virulent and temperate phages in AS and biofilm metagenomes. For AS samples, the proportion of phages represents the average of four samples

By leveraging the phage catalog, we performed an extensive analysis of the taxonomic classification of phages recovered from this system. Given the absence of a universal marker gene in phages, microbial-style marker-based taxonomy classification is not feasible [44]. Furthermore, considering the substantial diversity and novelty of phages in biological systems, methods suitable for the annotation and classification of novel phages are required. Therefore, in this study, both the gene-sharing network clustering algorithm (vConTACT2 [44]) and a graph convolutional network-based deep learning classifier (PhaGCN2 [45]) were utilized. Gene-sharing network clustering was performed by calling the proteins encoded by phages and taxonomically known reference phage genomes from the Viral RefSeq (Fig. 3a). After removing the reference phage genome-only clusters, the results showed that a total of 8596 vOTUs were clustered into 2538 viral clusters (VCs) with two or more members (Additional file 2: Table S6); this resulted in approximately 46% of vOTUs being assigned to a VC. By examining the presence of reference genomes in the VCs, only 59 VCs (225 vOTUs) could be assigned to a known viral family, and about 86% of these annotated VCs were classified into the family of *Siphoviridae*, *Myoviridae*, and *Podoviridae* (Fig. 3a). The lower assignment rate indicated that this system harbored a substantial number of unexplored phages. For the classification using PhaGCN2, which has enhanced the resolution of taxonomic classification of phages, similar constitutes for taxonomy profiling were obtained (Fig. 3a and Additional file 2: Table S7). Additionally, lifestyle prediction results indicated that about 62% of the reconstructed phages are lytic (Fig. 3a and Additional file 2: Table S8).

Metaviromic and bulk metagenomic data from these two sample types were used to profile the distribution and diversity of phage communities in biofilm and AS (Additional file 2: Table S9), respectively. Apparent dissimilarities in phage communities between biofilm and AS were revealed by PCoA analyses (Additional file 1: Fig. S5a, b). Only 4867 vOTUs and 1817 vOTUs were shared among all samples in the metavirome and metagenome, respectively (Fig. 3b, c); meanwhile, a large number of vOTUs were shared amongst AS samples, emphasizing the distinct phage composition between AS and biofilm. Moreover, the phage diversities in AS and biofilm were also compared. In metaviromes, higher Shannon and Simpson diversity of biofilm were observed, and minor distinctions in the diversity of AS samples were demonstrated (Additional file 1: Fig. S5c, d). For the metagenomes, the Shannon and Simpson indexes were not consistent with the result of metaviromes; lower diversity indices were observed in biofilm (Additional file 1: Fig. S5c, d). Lower phage coverage in both biofilm metavirome and metagenome was observed, although higher diversity was observed in the biofilm metavirome (Fig. 3d, e). Notably, both in the metavirome and metagenome, these samples displayed little differences in virulent and temperate phage fractions (Fig. 3d, e).

### Tight-connected prokaryotic hosts and phages

The integration of conventional in silico approaches and Hi-C method reported that 1190 vOTUs could be linked to 335 prokaryotes reconstructed from this system. CRISPR spacer-based searching reported 177 host-phage pairs. Homology and tRNA sequence searching found 393 and 69 host-phage pairs, respectively. Furthermore, the Hi-C signal was able to find as many as 817 host-phage pairs, which significantly improved the resolution of host-phage relationships. After summarizing the host-phage pairs assigned by these four methods, a total of 1340 host-phage pairs were ultimately obtained (Additional file 1: Fig. S6 and Additional file 2: Table S10). These putative hosts spanned 23 bacterial phyla, with *Pseudomonadota* and *Chloroflexota* being the most frequently predicted hosts (Additional file 1: Fig. S7). More intriguingly, 77 vOTUs were predicted to infect *Patescibacteria*, which are normally with small genome sizes. In addition, 170 vOTUs were linked to *Planctomycetota*, including abundant anammox. Other nitrifiers were also found to be infected by phages in this system, such as AOB, comammox, and NOB.

**Fig. 4:**
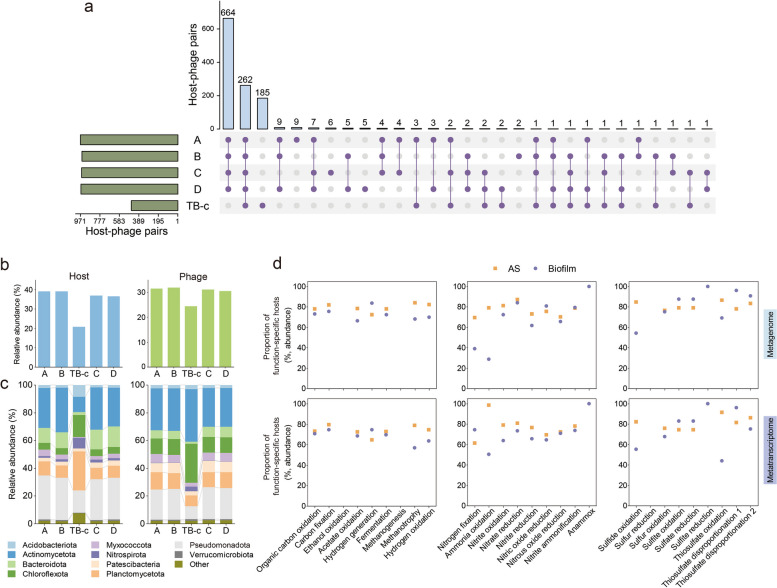
Host-phage interactions and potential metabolic functions of hosts. **a** The upset chart shows the number of host-phage pairs and their sharedness in each sample. **b** The proportion of predicted hosts and their associated phages within the whole prokaryotic and phage communities, respectively. **c** The relative abundance of predicted hosts and their associated phages at the phylum level. For phage, the coverage of phage with a host affiliated with the same phylum was summed and divided by the summed coverage of phages with putative hosts. **d** The proportion of function-specific hosts contributing to the overall metabolic capacity or activity of specific metabolic functions in the metagenome (upper panel) and metatranscriptome (lower panel). The proportion was calculated by dividing the abundance or activity of function-specific hosts by the abundance or activity of function-specific prokaryotes in the system

Upon obtaining the host-phage pairs collection, the prevalence of host-phage pairs in both AS and biofilm was explored using bulk metagenomes. A host-phage pair in a bulk metagenome will be considered present if the phage coverage exceeds 0.1 and the host’s relative abundance is over 0.01% for that specific pair. Results demonstrated that more than 660 host-phage pairs were exclusively observed in AS samples, while 185 host-pairs occurred solely in biofilm, emphasizing the distinct prokaryote-phage infection events between these two environments (Fig. 4a). In addition, it was found that approximately 24.5–31.9% (based on coverage) of the phages could be assigned to specific hosts, and the predicted hosts accounted for 21.1–39.0% of the entire microbial community (Fig. 4b). Notably, the distribution of phages was found to be in good agreement with that of their predicted prokaryotic hosts, based on the grouping results of host-phage pairs at the phylum level (Fig. 4c). This highly consistent trend in the appearance of hosts and their related phages suggests a strong connection between them. Furthermore, the distinct composition of the identified host between AS and biofilm can be anticipated based on the distinct host-phage pairs profile observed for both AS and biofilm. Importantly, the majority of these predicted hosts occupied more than 70% of the abundance of the prokaryotes with a specific metabolic capacity identified in this system, and they also represented the active members involved in a particular metabolic process, as indicated by the proportion of the transcriptional activities of these hosts (Fig. 4d). These results suggested that phages may play a crucial role in mediating the carbon, nitrogen, and sulfur cycling in this system by regulating the host metabolism.

### Auxiliary metabolic potential of phages

**Fig. 5:**
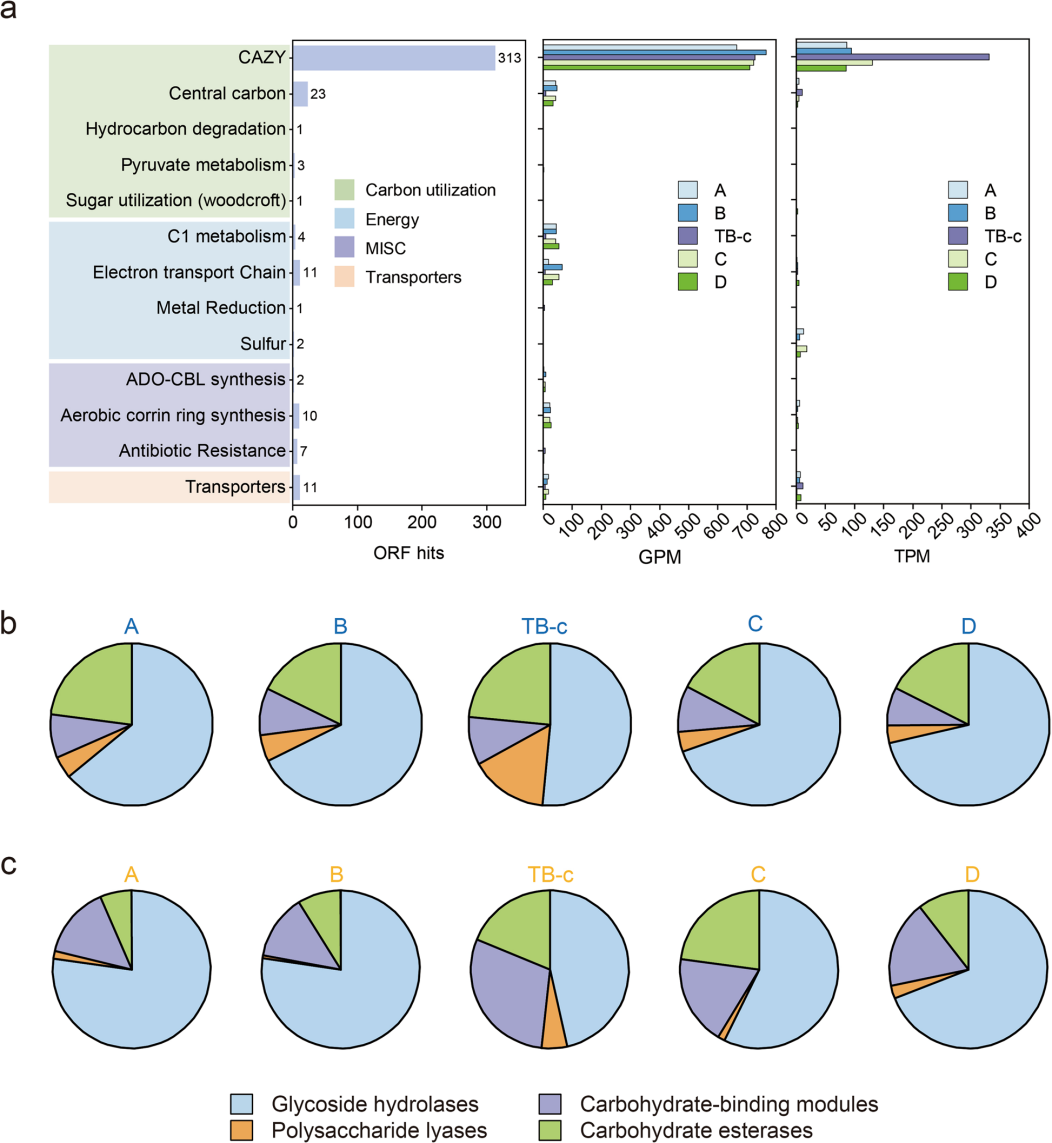
Abundance and activity of predicted AMGs. **a** The summary of predicted AMGs from reconstructed vOTUs, as well as their abundance (GPM) and expression (TPM) in AS and biofilm samples. **b** Proportions of genes (abundance) associated with various CAZymes. **c** Proportions of gene expression associated with various CAZymes

The auxiliary metabolic potential of reconstructed phage was also explored through the prediction and annotation of AMGs encoded by the phages. A total of 5253 phage genes were predicted as AMGs based on the DRAM-v annotation, of which 2301 could be obtained annotation information (Additional file 2: Table S11). Results indicated that most putative AMGs were assigned to the category related to carbon utilization, organic nitrogen metabolism, and miscellaneous (MISC) processes (Additional file 2: Table S11). Given the credibility of the prediction of their auxiliary function, genes related to glycosyl transferases, peptidase, amino acid, and information systems were excluded from subsequent discussion [48, 49]. Of these retained predicted AMGs involved in carbon utilization, 197 putative AMGs encoding glycoside hydrolases and 22 putative AMGs encoding polysaccharide lyases were found. Intriguingly, two AMGs capable of driving the assimilatory sulfate reduction and two AMGs involved in the urea cycle were predicted, respectively. Moreover, AMGs involved in central carbon, C1 metabolism, hydrocarbon degradation, and other modules were also identified in these phage genomes (Fig. 5a and Additional file 2: Table S11). We additionally noted that the difference between the annotated numbers of AMGs and the expected frequencies of AMGs (calculated based on the proportion of genes of each category in the reference databases) (Additional file 2: Table S12) was statistically highly significant (chi-square *p* < 0.001). This finding implies that the enrichment of certain categories is not solely the result of the database size.

To predict the potential function of AMGs in this system, we conducted further analysis on their abundance and expression levels. Results reveal that the gene abundance of AMGs associated with the carbohydrate-active enzymes (CAZymes) is relatively higher than that of other categories (Fig. 5a). Additionally, highly expressed genes belonging to CAZymes were observed in biofilm (Fig. 5a). Furthermore, we subdivided the modules belonging to this category and explored the abundance (Fig. 5b) and expression (Fig. 5c) of genes within these modules. Interestingly, different expression profiles were observed between biofilm and AS samples. In AS samples, genes related to glycoside hydrolases exhibited the highest expression, whereas in biofilm, a comparable expression of genes associated with glycoside hydrolases and carbohydrate-binding modules was observed. Furthermore, phage-mediated assimilatory sulfate reduction activity was observed in AS samples.

### Phage-host infection dynamics

**Fig. 6:**
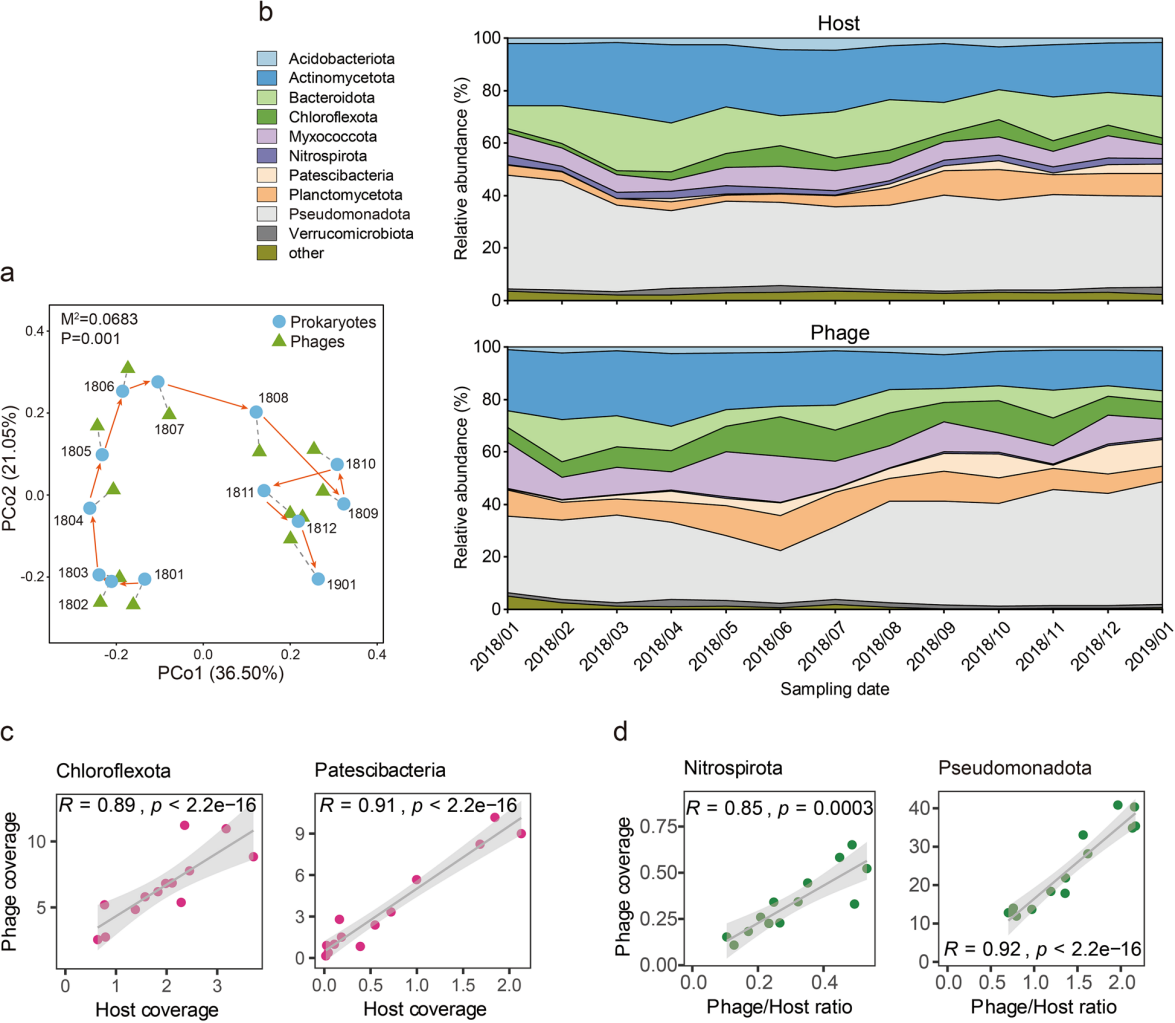
Temporal dynamics and infection feature of host-phage pairs. **a** Procrustes analysis of prokaryotic and phage communities over time. Prokaryotic and phage community members were represented by the reconstructed MAGs and vOTUs, respectively. The years 2018 and 2019 are denoted by the numbers 18 and 19, respectively, while the last two digits indicate the corresponding month. **b** Relative abundance of predicted hosts and associated phages over time, which was summarized at the phylum level. **c** Spearman correlation between host and phage coverage at the phylum level. **d** Spearman correlation between phage/host ratio and phage coverage at the phylum level. The shaded areas represent 95% confidence intervals

The dynamics of phage-host infection were explored by employing the abundance information of prokaryotes and phages calculated from the time-series AS metagenomic data (Additional file 2: Table S13, S14). First, we examined the dissimilarities in the overall prokaryotic and phage communities over time and investigated the relationships between these two communities using Procrustes analysis. Notably, Procrustes analysis revealed highly consistent trends in the changes for both communities, emphasizing that phage composition was strongly correlated (*M*^2^ = 0.0683, *p* = 0.001) with prokaryotic composition (Fig. 6a). Furthermore, we profiled the relative abundance of phages and their associated hosts at the phylum level, uncovering noticeable variations over time (Fig. 6b). For instance, the relative abundance of the *Planctomycetota* populations increased over time, while an opposite trend was observed for *Actinomycetota*. For phages, the relative abundance of *Planctomycetota*-related phages did not show similar trends as their hosts, while a consistent decrease trend was shown for *Actinomycetota*-related phages. Consistent fluctuation changes in related phages were demonstrated for the phyla of *Bacteroidota*, *Chloroflexota*, and *Myxococcota*. For the abundant phylum *Pseudomonadota*, their abundance remained stable over time, while the related phage increased. Based on these varying trends of prokaryotic hosts and phages, we speculate that host-phage may have various infection dynamics.

To explore the infection patterns of hosts and phages, we performed a correlation analysis using the Spearman correlation coefficient at the phylum level. Very strong positive correlations were observed for *Chloroflexota*, *Patescibacteria*, and their associated phages (Fig. 6c). Additionally, *Actinomycetota*, *Bacteroidota*, and *Myxococcota* and their related phages also exhibited strong correlations (Additional file 1: Fig. S8). These findings suggest that these phage populations increased or decreased in tandem with their hosts. However, no consistent or opposite variation trends were observed for other phyla. To further explore their potential interactions or phage infection mechanisms, we also examined the phage/host ratios to indicate infection events or phage replication activity over time. We observed variations in the phage/host ratios for different phyla during this period (Additional file 1: Fig. S9). According to correlation results, a very strong correlation between the phage/host ratio and phage coverage for *Nitrospirota* and *Pseudomonadota* was observed (Fig. 6d). Significant correlations were also found for the phyla of *Acidobacteriota*, *Bacteroidota*, and *Myxococcota* (Additional file 1: Fig. S10). This implies that the increase or decrease of phages was not solely determined by host abundance but was influenced by changes in the phage/host ratio, that is, their infection or replication capability within host cells.

## Discussion

In this study, the distribution and activities of functional microbes were investigated by integrating metagenomics and metatranscriptomics. Distinct microbial community compositions were observed between AS and carrier biofilm, with abundant eukaryota and archaea present in the biofilm. High-quality genomes recovered through hybrid assembly revealed that this system harbored numerous novel organisms not represented in existing databases, including diverse and abundant nitrifiers involved in the nitrogen cycle and archaea populations affiliated with the DPANN superphylum. Notably, the unique characteristics of this system allow for distinct distribution patterns and varying activities of functional microbes. For instance, AOB, NOB, and comammox were found in both the biofilm and AS, while anammox bacteria were detected only in the biofilm with high abundance. The substrate and oxygen gradients within this system may explain this distribution [60, 61]. Regarding the denitrification process, higher activities for nitrite, nitric oxide, and nitrous oxide reduction were observed in AS samples, particularly in tank A, the anoxic tank in this treatment system. Higher transcriptional activities of genes for nitrate reduction were observed in the biofilm, suggesting that the nitrite generated during this process may actuate the anammox reactions in the biofilm, emphasizing the cross-feeding within this system [6]. Collectively, the biochemical characteristics of microorganisms play a crucial role in shaping their spatial distribution, which subsequently influences their accessibility to various substrates. Further studies are needed to investigate the metabolite exchange mechanisms among different populations, as well as the strategies employed for their preservation.

The phage community, similar to the prokaryotic community, displays notable differences between AS and biofilm samples. A considerable number of unique phages were enriched and identified in both AS and biofilm samples, with these variations also being evident in the corresponding bulk metagenomes. This finding is consistent with the conventional wisdom that phages and hosts are highly dependent and that most phages have a narrow host range [55]. In addition to the distribution of hosts leading to differences in phages, the characteristics of their surrounding environment will also affect their distribution and dispersal. The lower coverage in the biofilm suggested differences in phage density. Previous studies have suggested that the microbial communities within biofilms are less susceptible to phage infections due to the inhibition of phage transport into the biofilm [62, 63]. This limitation makes it less likely for phages to infect bacteria located farther away within the biofilm. In contrast, AS slurry is more favorable for the dispersal and infection of phages, which could account for their high density in AS compared to biofilm.

The high-resolution analysis of host-phage relationships using the Hi-C technique enables us to gain a comprehensive understanding of the potential ecological roles of phages by linking them to functional prokaryotes. Notably, the high proportion of infected prokaryotic hosts involved in various metabolic functions highlighted the potential vital roles that phages have in influencing this system. In addition to regulating the host mortality and reprogramming host metabolism, which in turn impacts the metabolic functions, phages also appear to impact system function through auxiliary metabolism, as suggested by the AMGs encoded in their genomes. The abundance and activity of AMGs encoding glycoside hydrolases and polysaccharide lyases suggest that phages contribute to the breakdown of complex carbohydrates and polysaccharides. This could potentially facilitate microbial carbon degradation and utilization, ultimately influencing carbon cycling within this system [64, 65]. The high expression of carbohydrate-binding modules might aid in the binding of glycoside hydrolases to the substrate, particularly in biofilm, subsequently promoting the hydrolysis of complex carbohydrates [66]. Furthermore, the presence of sulfur auxiliary metabolism in recovered phages was observed, emphasizing their potential roles in the associated cycling processes. Similar findings of the potential auxiliary metabolism of phages have been reported in various systems [48, 49, 64, 67, 68].

Time-series data analysis enables us to explore the dynamics of prokaryotic and phage communities over time. By leveraging identified host-phage pairs, correlation analyses between phages and their associated hosts lead us to speculate that phage-host infection dynamics. Our findings reveal a strong correlation between the abundance of phages infecting specific phyla, such as *Chloroflexota* and *Patescibacteria*, and the abundance of their respective hosts. However, for phages infecting other phyla like *Nitrospirota* and *Pseudomonadota*, the phage/host ratio determines their abundance, with infection rates or reproduction abilities changing alongside their associated hosts. Previous studies have highlighted that the phage/host ratio is an important factor reflecting the host-phage relationships [65, 69, 70]. Importantly, phage/host ratio varies across different ecosystems and environmental conditions have been revealed [65, 71]. The varied lineage-specific phage/host ratios imply that the shifting in biotic and/or abiotic parameters may affect the phage-host infection dynamics, subsequently affecting microbial responses to the altered environmental conditions [65]. For the phylum of *Nitrospirota*, their core members are NOB and comammox, the altered phage/host ratio imply a potentially vital role of phages in influencing nitrogen cycling within this system. Additionally, the phage/host ratio may be indicative of phage activity, as actively replicating phages would exhibit higher genome coverage than their hosts [70]. In this system, a higher and varied phage/host ratio was observed for the phyla *Planctomycetota*, *Chloroflexota*, and *Myxococcota*, suggesting that the phages associated with these phyla are more active (Additional file 1: Fig. S9). Microorganisms affiliated with these phyla represent a considerable proportion of the prokaryotic community and participate in various metabolic processes. This observation further implies that phages may have a vital impact on the transformation and cycling of substances within the system. Collectively, these findings highlight that not only environmental conditions might influence prokaryotes, but bacteriophage infections also play a role in shaping prokaryotic communities. However, further research is needed to understand how phage infections are affected by environmental conditions and how these infections in turn impact the response of hosts to the environmental conditions.

The current life strategies of phages encompass the “kill-the-winner” and “piggyback-the-winner” models [72, 73]. The “kill-the-winner” model emphasizes the lytic life stage of viruses, suggesting that when a host is actively growing and its population numbers are increasing, lytic infections will be favored over lysogenic infections. In contrast, the “piggyback-the-winner” model posits that lysogeny is favored when hosts are abundant [72]. In this study, the fractions of lytic or temperate phages are not well correlated with host abundance (Additional file 1: Fig. S11), suggesting the existing viral infestation models may not be suitable for explaining the complex system. One potential explanation is that the metagenome predominantly captures viable or infectable cells along with their intracellular phages from AS samples [70]. Meanwhile, lysed cells and released phages may not be readily available for detection as they are released into the surrounding fluid. Moreover, a recent study has highlighted that phage adsorption and entry into host cells do not equate to full completion of the lytic cycle owing to cellular defense systems [74]; therefore, in-depth investigation into the infection mechanisms may provide valuable insights and help address this question. Furthermore, Berg et al. have suggested that lytic and lysogenic viruses can readily co-infect the same host population and that host strain-level diversity might be an important factor controlling virus-host dynamics including lytic/lysogeny switch [72]. Therefore, investigating the dynamics of phages at the host strain level is of great importance, especially for the important functional microbes involved in the pollutant removal process.

## Conclusions

In conclusion, our study provides a comprehensive understanding of the relationships between prokaryotic and phage communities in this hybrid biological system. Distinct distributions of prokaryotic and phage communities were revealed between AS and biofilm samples. Additionally, a tremendous diversity of phages was depicted, with a higher abundance of phages found in AS. The Hi-C approach substantially augments the linking of prokaryotes and phages, highlighting that phages may impact the ecosystem function by regulating host mortality and metabolism, as well as influence substance cycling through auxiliary metabolism. Furthermore, the integration of these newly established host-phage pairs with time-series metagenomic data unveiled phage-host infection dynamics over time, further emphasizing the potentially crucial impact of phages on system performance and stability. Gaining a deep understanding of the relationship between biotic and/or abiotic parameters and infection dynamics represents an important and intriguing area for further research.

### Supplementary Information

Additional file 1: Characteristics of WWTP. Phage enrichment processes. Assembly of putative phage contigs. Hi-C library sequencing and subsequent host-phage linkage matching. Fig. S1. The diagram of the different sampling tanks. Fig. S2. The relative abundance of bacteria, archaea, and eukaryota in AS and biofilm. Fig. S3. The phylogenetic tree of the retrieved 453 bacterial MAGs. Fig. S4. Microorganisms involved in carbon, nitrogen, and sulfur cycling in this system. Fig. S5. Comparison of phage community diversity between activated sludge and carrier biofilm. Fig. S6. Host-phage associations were predicted using multiple approaches, including Hi-C sequencing (solid line), homology alignment (double solid line), spacer searching (dashed line), and tRNA searching (arrows). Fig. S7. Phylogenetic distribution of bacterial hosts at the phylum level. Fig. S8. Spearman correlation analysis between host and phage coverage at the host phylum level. Fig. S9. Variations in the phage/host ratio over time at the phylum level. Fig. S10. Spearman correlation analysis between phage/host ratio and phage coverage at the host phylum level. Fig. S11. Spearman correlation analysis between host coverage and virulent phage ratio at the host phylum level.

Additional file 2: Table S1. Water parameters of different tanks. Table S2. Characteristics of the 454 reconstructed MAGs. Table S3. The relative abundance and transcriptional activity of MAGs in activated sludge and biofilm samples. Table S4. The detailed information of vOTUs predicted from different assemblies. Table S5. CheckV result of the recontructed vOTUs. Table S6. Viral clusters consist of members equal to or greater than two. Table S7. PhaGCN2 classification result for phage genomes. Table S8. Prediction results of phage lifestyle. Table S9. Phage coverage in in-situ samples and enriched-VLP samples. Table S10. Predicted host-phage linkages using four different methods. Table S11. AMGs identified in reconstructed phage genomes using DRAM-v. Table S12. Filtered annotated and expected AMG numbers. Table S13. Prokaryote coverage calculated from a 13-month time-series AS metagenomic data. Table S14. Phage coverage calculated from a 13-month time-series AS metagenomic data.

## Data Availability

All sequencing data generated in this study are deposited in the NCBI Sequence Read Archive (SRA) database under the BioProject ID PRJNA1012837 (https://www.ncbi.nlm.nih.gov/bioproject/?term=PRJNA1012837). Additionally, the MAGs recovered from this study can be accessed using the same project accession number. Other relevant information is included in the manuscript and supporting files.

## Abbreviations

AMG: Auxiliary metabolic gene
Anammox: Anaerobic ammonium oxidation
ANI: Average nucleotide identity
AOB: Ammonia-oxidizing bacteria
AS: Activated sludge
Comammox: Complete ammonia oxidation
Gb: Gigabase
HCBHA: Haplotype-resolved hierarchical clustering-based hybrid assembly
HMBBR: Hybrid moving bed biofilm reactor
MAG: Metagenome-assembled genome
NOB: Nitrite-oxidizing bacteria
ORF: Open reading frame
VC: Viral cluster
VLPs: Virus-like particles
vOTU: Viral operational taxonomic unit
WWTPs: Wastewater treatment plants
