# Supplementary material for: Distributions, interactions, and dynamics of prokaryotes and phages in a hybrid biological wastewater treatment system
Source: Microbiome. 2024 Jul 22;12:134. doi: 10.1186/s40168-024-01853-6 (PMC11265110; doi:10.1186/s40168-024-01853-6)
Supplement: Supplementary file 2 — Additional file 1: Characteristics of WWTP. Phage enrichment processes. Assembly of putative phage contigs. Hi-C library sequencing and subsequent host-phage linkage matching. Fig. S1. The diagram of the different sampling tanks. Fig. S2. The relative abundance of bacteria, archaea, and eukaryota in AS and biofilm. Fig. S3. The phylogenetic tree of the retrieved 453 bacterial MAGs. Fig. S4. Microorganisms involved in carbon, nitrogen, and sulfur cycling in this system. Fig. S5. Comparison of phage community diversity between activated sludge and carrier biofilm. Fig. S6. Host-phage associations were predicted using multiple approaches, including Hi-C sequencing (solid line), homology alignment (double solid line), spacer searching (dashed line), and tRNA searching (arrows). Fig. S7. Phylogenetic distribution of bacterial hosts at the phylum level. Fig. S8. Spearman correlation analysis between host and phage coverage at the host phylum level. Fig. S9. Variations in the phage/host ratio over time at the phylum level. Fig. S10. Spearman correlation analysis between phage/host ratio and phage coverage at the host phylum level. Fig. S11. Spearman correlation analysis between host coverage and virulent phage ratio at the host phylum level. [file 40168_2024_1853_MOESM1_ESM.docx]

**Supplementary Information**

**Distributions, interactions, and dynamics of prokaryotes and phages in a hybrid biological wastewater treatment system**

Dou Wang^1^, Lei Liu^1^, Xiaoqing Xu^1^, Chunxiao Wang^1^, Yulin Wang^1^, Yu Deng^1^, Tong Zhang^1,2,3*^

^1^Environmental Microbiome Engineering and Biotechnology Laboratory, Center for Environmental Engineering Research, Department of Civil Engineering, The University of Hong Kong, Hong Kong SAR, China

^2^School of Public Health, The University of Hong Kong, Hong Kong SAR, China

^3^Macau Institute for Applied Research in Medicine and Health, Macau University of Science and Technology, Macau SAR, China

*Corresponding author.

E-mail: zhangt@hku.hk; Tel. 852-28578551; Fax 852-25595337.

This document includes

**Supplementary Methods**

Characteristics of WWTP

Phage enrichment processes

Assembly of putative phage contigs

Hi-C library sequencing and subsequent host-phage linkage matching

**Supplementary Figures**

**Fig. S1** The diagram of the different sampling tanks.

**Fig. S2** The relative abundance of bacteria, archaea, and eukaryota in AS and biofilm

**Fig. S3** The phylogenetic tree of the retrieved 453 bacterial MAGs.

**Fig. S4** Microorganisms involved in carbon, nitrogen, and sulfur cycling in this system.

**Fig. S5** Comparison of phage community diversity between activated sludge and carrier biofilm.

**Fig. S6** Host-phage associations were predicted using multiple approaches, including Hi-C sequencing (solid line), homology alignment (double solid line), spacer searching (dashed line), and tRNA searching (arrows).

**Fig. S7** Phylogenetic distribution of bacterial hosts at the phylum level.

**Fig. S8** Spearman correlation analysis between host and phage coverage at the host phylum level.

**Fig. S9** Variations in the phage/host ratio over time at the phylum level.

**Fig. S10** Spearman correlation analysis between phage/host ratio and phage coverage at the host phylum level.

**Fig. S11** Spearman correlation analysis between host coverage and virulent phage ratio at the host phylum level.

**Supplementary Methods**

**Characteristics of WWTP**

The full-scale hybrid MBBR is part of Stanley Sewage Treatment Works (Stanley STW) located in Hong Kong, which is a secondary sewage treatment works built in caverns. It serves a population of over 27,000 and currently produces around 9,000 cubic meters of sewage per day. The treatment system under study is comprised of four compartments. The initial compartment functions as an anoxic denitrification zone, followed by a compartment containing activated sludge and carrier materials (25-millimeter diameter multi-faceted plastic particles) operating as a hybrid MBBR. Subsequently, two aerobic compartments without carriers are utilized (refer to Fig. S1 for details).

**Phage enrichment processes**

The initial step involves releasing phage particles into the liquor and separating the biomass and liquor. For the activated sludge sample, a 15-minute sonication was performed to detach viruses, followed by centrifugation at 4500 rpm for 20 minutes to collect supernatants. For the carrier sample, the biofilm was first brushed off, and then the biofilm slurry was homogenized using glass beads at 150 rpm for two hours. After homogenization, the biofilm slurry was divided into 50 mL tubes and sonicated for 3 minutes (interrupted by 30 seconds of manual shaking each minute), and centrifuged at 4500 rpm for 20 minutes to separate biomass particles. Supernatants were collected, and the biomass pellets from the initial extraction were resuspended in deionized water. The procedure was repeated twice, and supernatants from these three extractions were combined and subjected to subsequent processes. The following steps include phage particle enrichment and purification processes, which can be found in our previous works [1, 2]. In brief, supernatants from the previous step were filtered through 0.45 μm membranes (Advantec MFS, USA) and 0.22 μm Sterivex filters (Merck Millipore, USA) to remove cellular debris and other contaminants. The filtrate was iron-chloride flocculated, collected on 0.22 μm polycarbonate filters (Merck Millipore, USA), and resuspended in an oxalate solution. PEG was subsequently added to precipitate viruses. To eliminate the contamination of eukaryotic, prokaryotic, and extracellular nucleic acids, Turbo DNase (Thermo Fisher Scientific, USA) was added to remove unencapsidated nucleic acids. Liquor samples with enriched viral-like particles (VLPs) were used for the subsequent extraction procedure.

**Assembly of putative phage contigs**

Three different assembly approaches were used in this study, including short-read assembly, Flye assembly, and OPERA-MS assembly. For short-read assembly, the five enriched metaviromes were co-assembled using metaSPAdes (v3.10.1) [3] with default settings to get the candidate contigs for phage sequence prediction. For the Flye assembly process, Porechop (v0.2.4) (<https://github.com/rrwick/Porechop>) was employed to remove the adapter sequence of based-called Nanopore reads. The filtered long reads were then assembled using Flye (v2.9-b1768) [4] with the “--meta” option, followed by mapping of the short reads generated from VLPs-enriched samples to the resulting contigs using bwa (v0.7.17-r1188). Mapped reads were then used for error correction through Pilon (v1.24) [5], and the polishing process underwent three rounds. Finally, the OPERA-MS (v0.8.3) [6] assembler was also used for the viral contigs construction. The contigs from the short-read assembly were combined with filtered long reads and then overlayed with long-read information to construct an assembly graph of all contigs.

**Hi-C library sequencing and subsequent host-phage linkage matching**

The Hi-C library was prepared using the ProxiMeta Hi-C v4.0 Kit from Phase Genomics, following the guidelines provided by the manufacturer [7]. Briefly, the crosslinked AS and biofilm samples were simultaneously digested using the Sau3AI and MlucI [8] restriction enzymes. Biotinylated nucleotides were used to proximity-ligate the digested fragments, producing chimeric molecules that consisted of fragments from various genome regions that were physically close *in vivo*. The proximity-ligated DNA molecules were isolated using streptavidin beads and processed into an Illumina-compatible sequencing library. Sequencing was performed on the Illumina NovaSeq platform, generating PE150 read pairs.

After obtaining the Hi-C data, the Hi-C reads were then aligned to the assembly containing both phage and prokaryotic contigs, adhering to the Hi-C kit manufacturer's recommendations ([Aligning and QCing Phase Genomics Hi-C Data](https://phasegenomics.github.io/2019/09/19/hic-alignment-and-qc.html)). In brief, BWA-MEM Version 0.7.17 [9] was utilized for alignment, specifying the -5SP options, while maintaining all other options at their default settings. SAMBLASTER Version 0.1.24 [10] was used to flag PCR duplicates, which were subsequently excluded from the analysis. Alignments were then filtered with samtools Version 1.17 [11] using the -F 2304 filtering flag to remove non-primary and secondary alignments. The ProxiMeta platform [12] was then used to perform host-finding.

**Supplementary Figures**


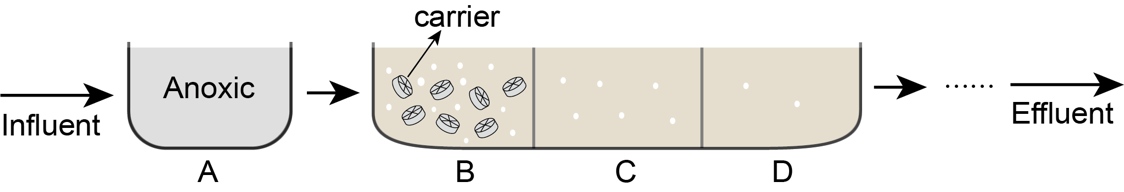


**Fig. S1** The diagram of the different sampling tanks.


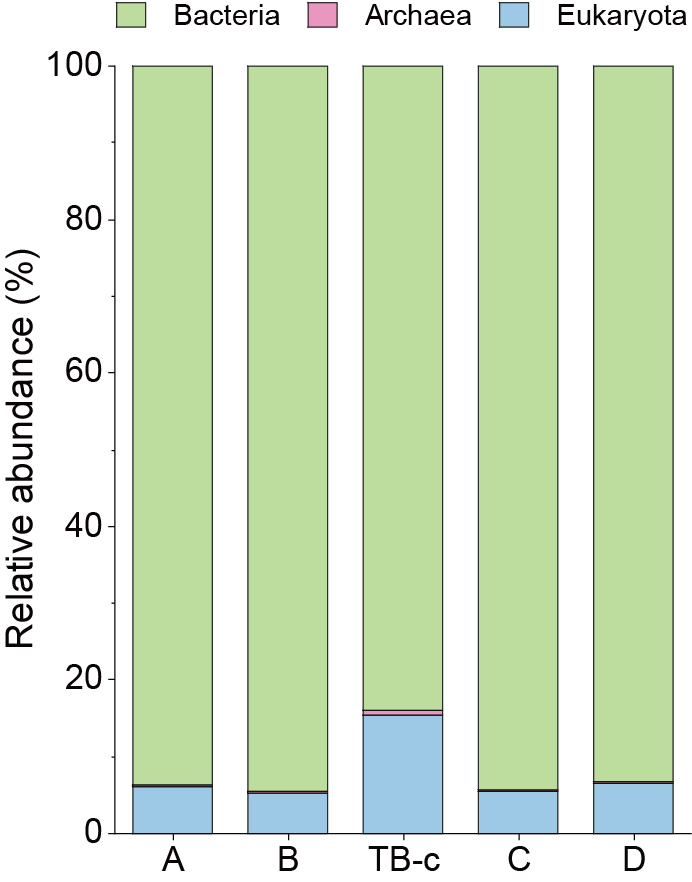


**Fig. S2** The relative abundance of bacteria, archaea, and eukaryota in AS and biofilm, which was profiled by mapping the metagenomic 16S and 18S rRNA gene reads to the SILVA SSU rRNA reference database.


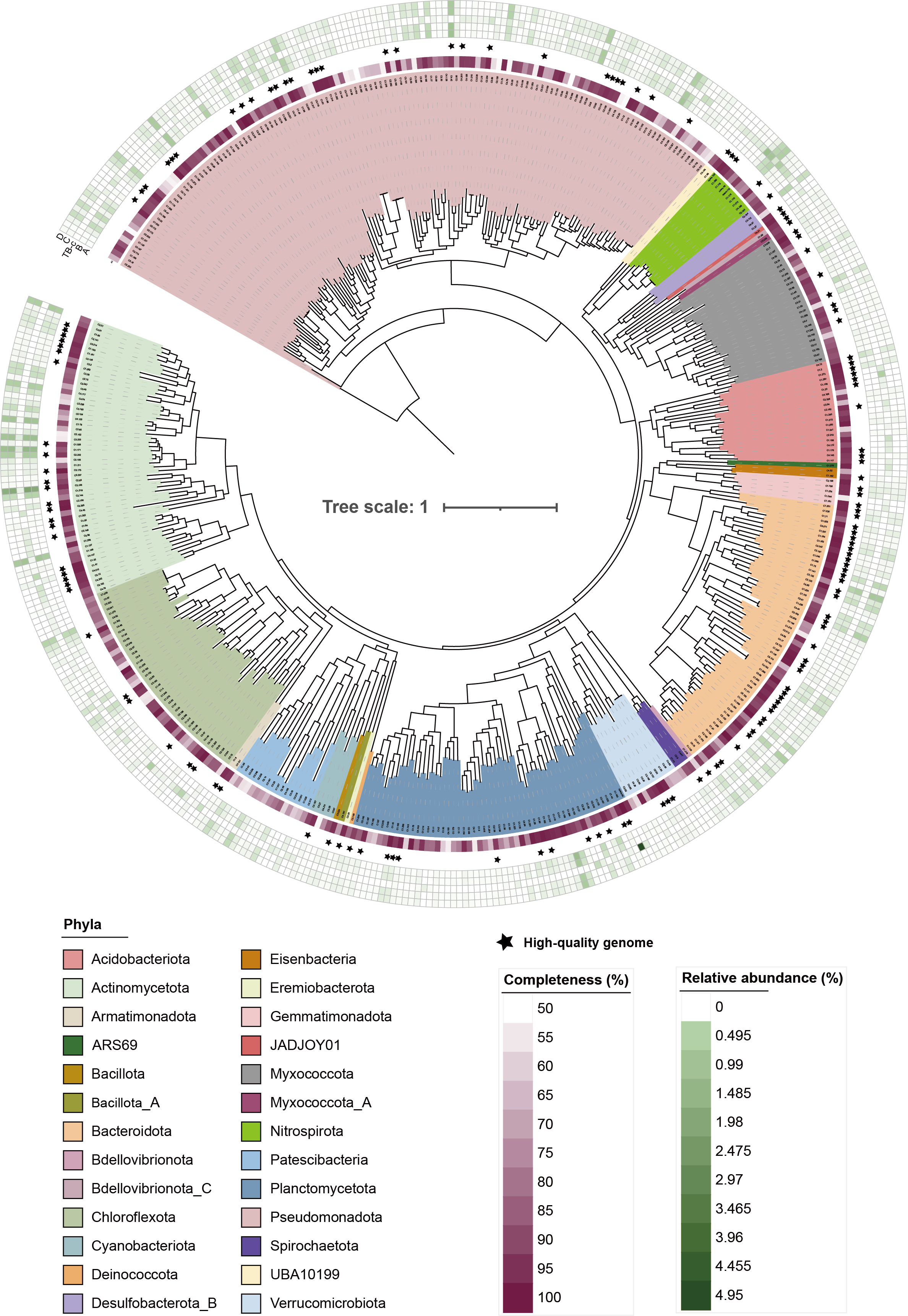


**Fig. S3** The phylogenetic tree of the retrieved 453 bacterial MAGs. The retrieved single archaeal MAG was not included in this tree. The genomes are shaded by phylum. The inner circular heatmap represents the genome completeness, and the outer circular heatmaps (inner-to-outer circular heatmap) indicate the relative abundance of individual MAGs in A, B, TB-c, C, and D metagenomes. Stars represent high-quality genomes classified based on the criteria defined by MIMAG standards.


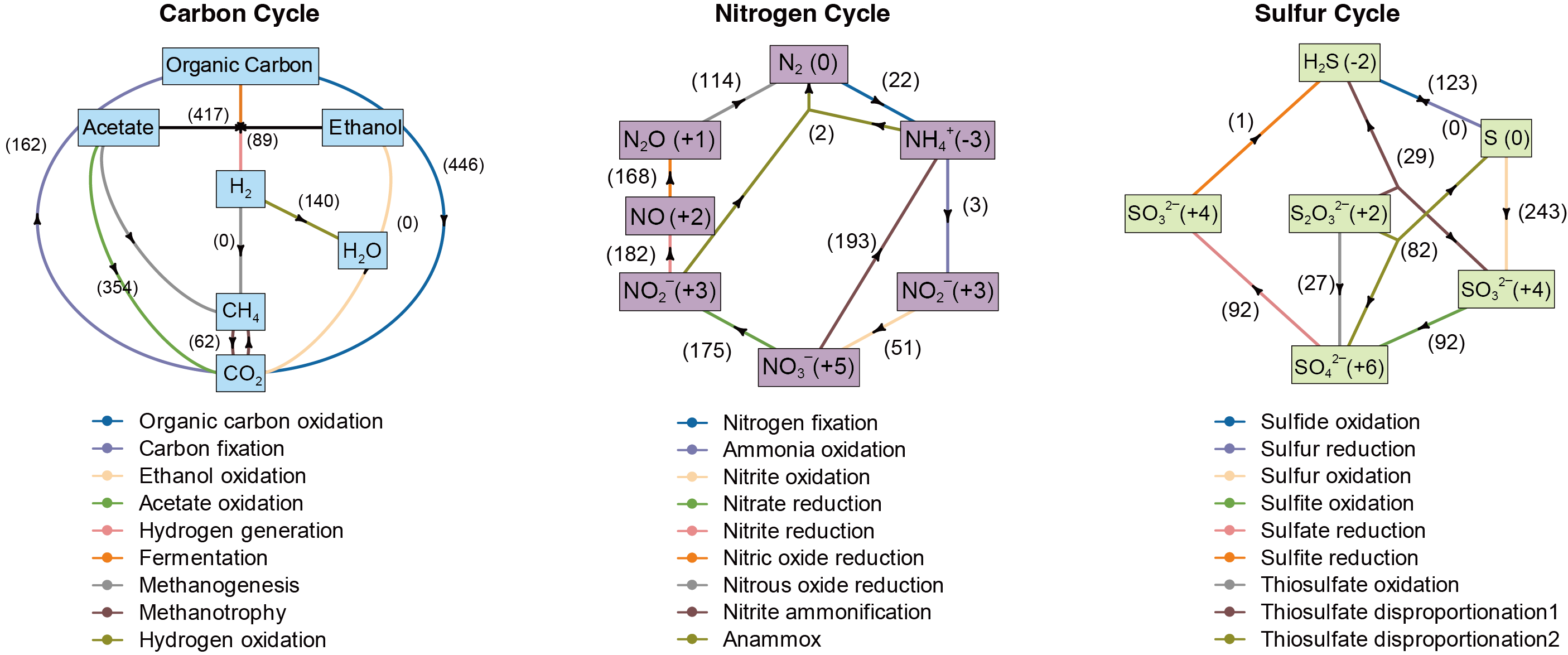


**Fig. S4** Microorganisms involved in carbon, nitrogen, and sulfur cycling in this system. The numbers in parentheses indicated the number of genomes recovered capable of carrying out that metabolic step.


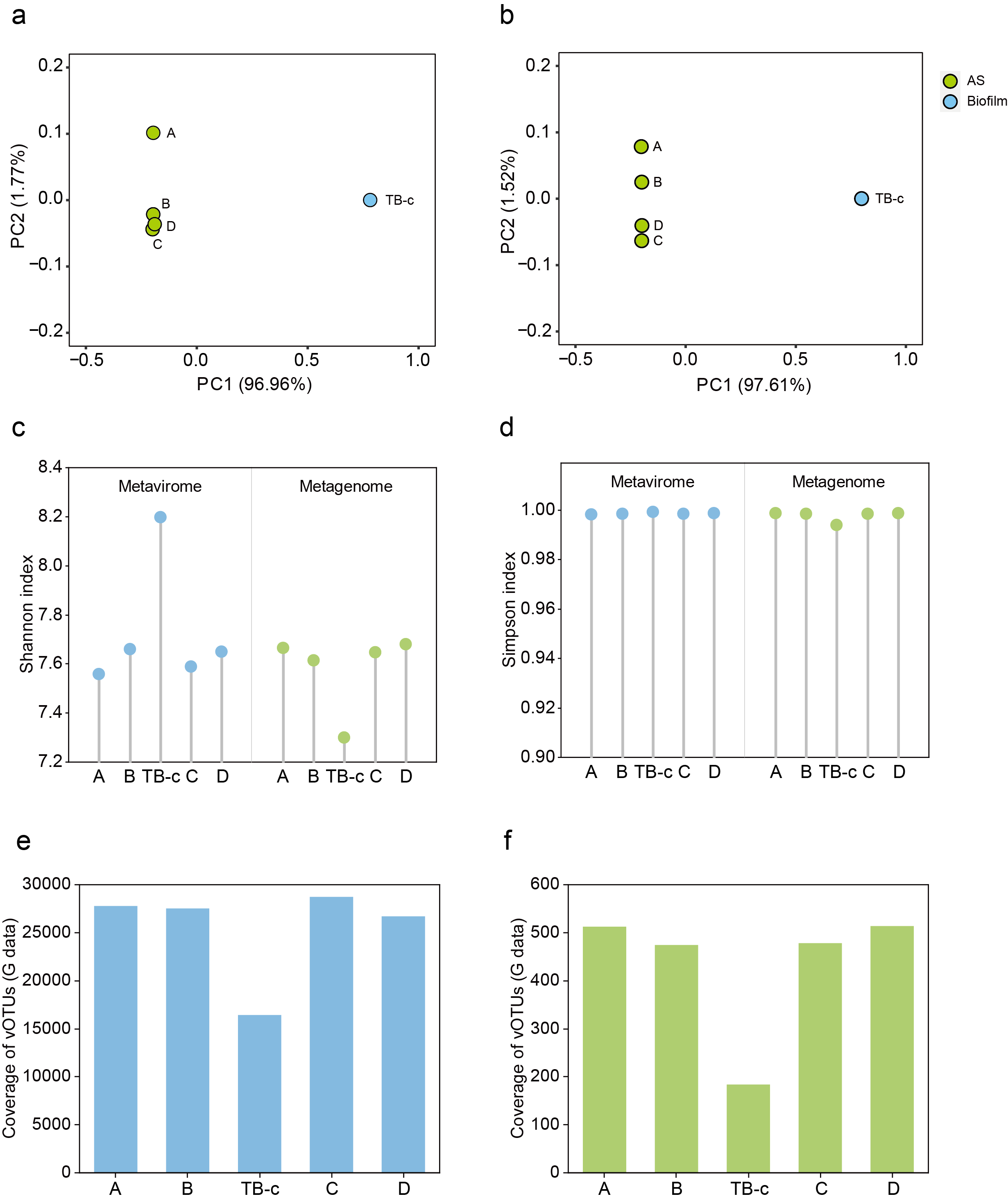


**Fig. S5** **Comparison of phage community diversity between activated sludge and carrier biofilm**. **a** Principal coordinate analysis (PCoA) based on Bray–Curtis dissimilarities calculated from phage coverage showing compositional differences of phage communities in metaviromes. **b** Principal coordinate analysis (PCoA) based on Bray–Curtis dissimilarities calculated from phage coverage showing compositional differences of phage communities in metagenomes. **c** Shannon diversity of phage communities in metaviromes (blue) and metagenomes (green). **d** Simpson diversity of phage communities in metaviromes (blue) and metagenomes (green).


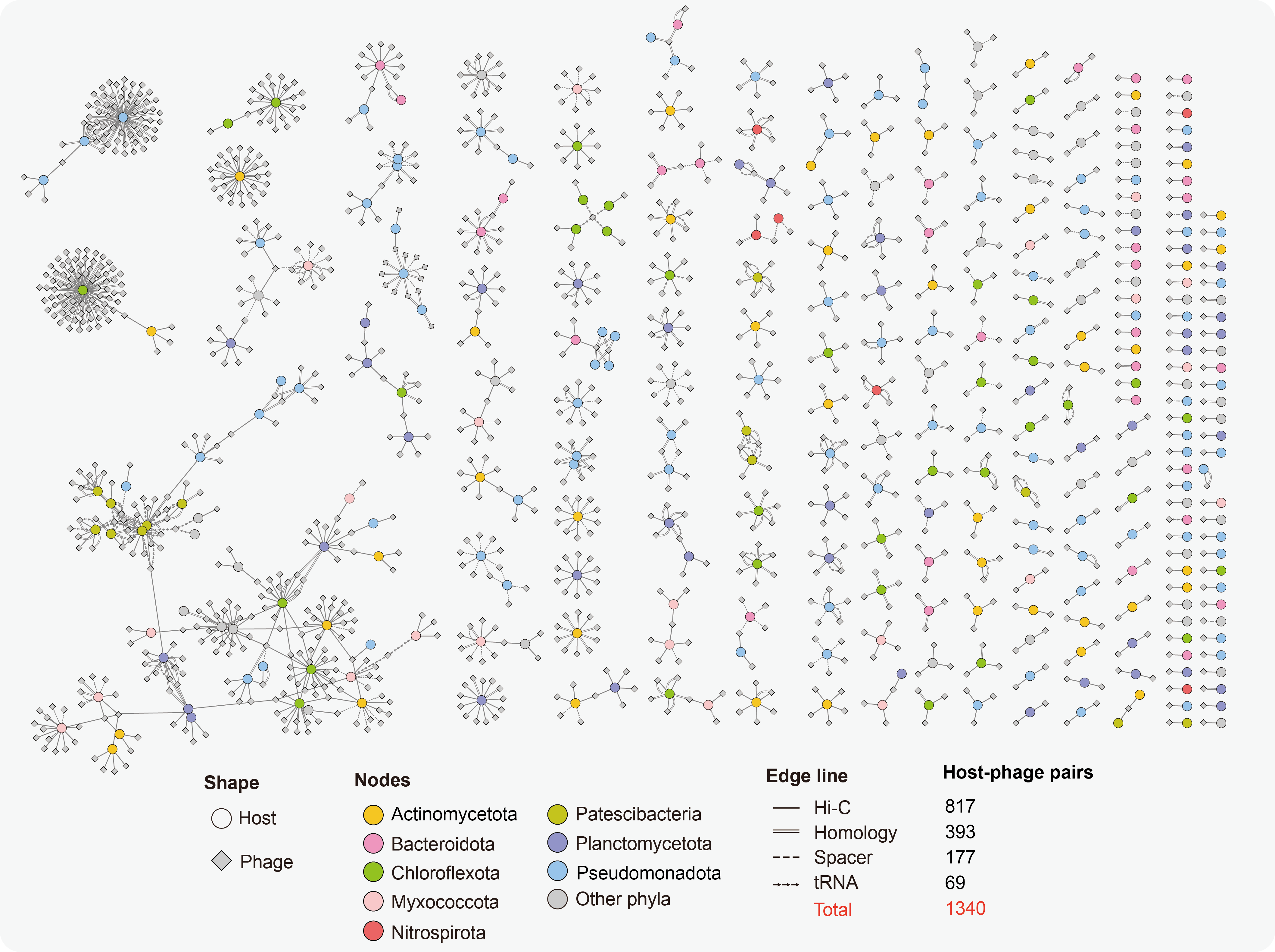


**Fig. S6** Host-phage associations were predicted using multiple approaches, including Hi-C sequencing (solid line), homology alignment (double solid line), spacer searching (dashed line), and tRNA searching (arrows). Circles represent the predicted hosts, and the colors of the circles indicate the phyla of the hosts.


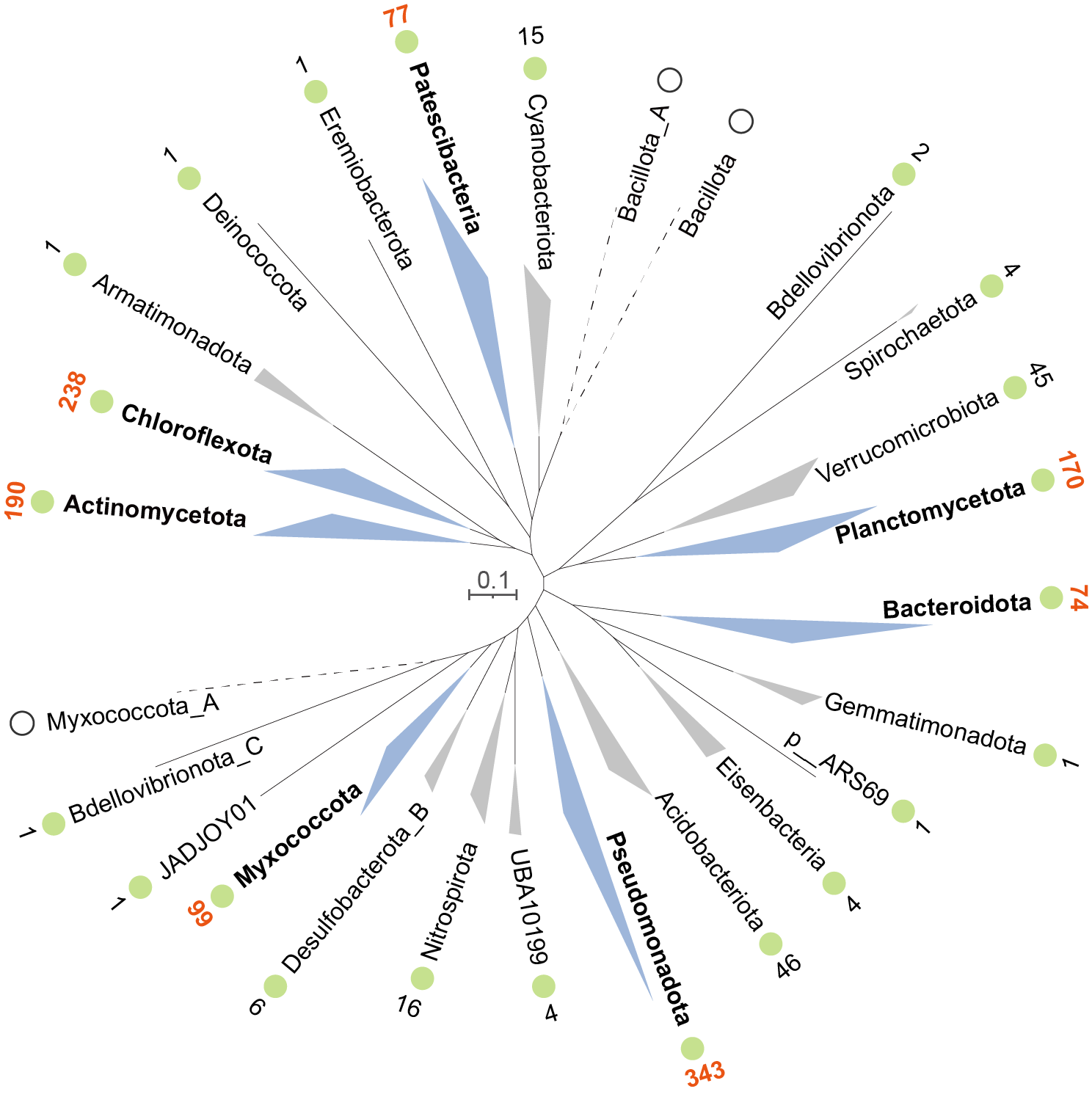


**Fig. S7** Phylogenetic distribution of bacterial hosts at the phylum level. The phylogenetic tree was inferred based on a concatenated alignment of 120 bacterial single-copy marker genes. The green circle indicates that the bacteria affiliated with this phylum were predicted to be the hosts of phages. The number near the circle represents the number of phages that could infect this phylum. The white circle represents the phylum without associated phages.


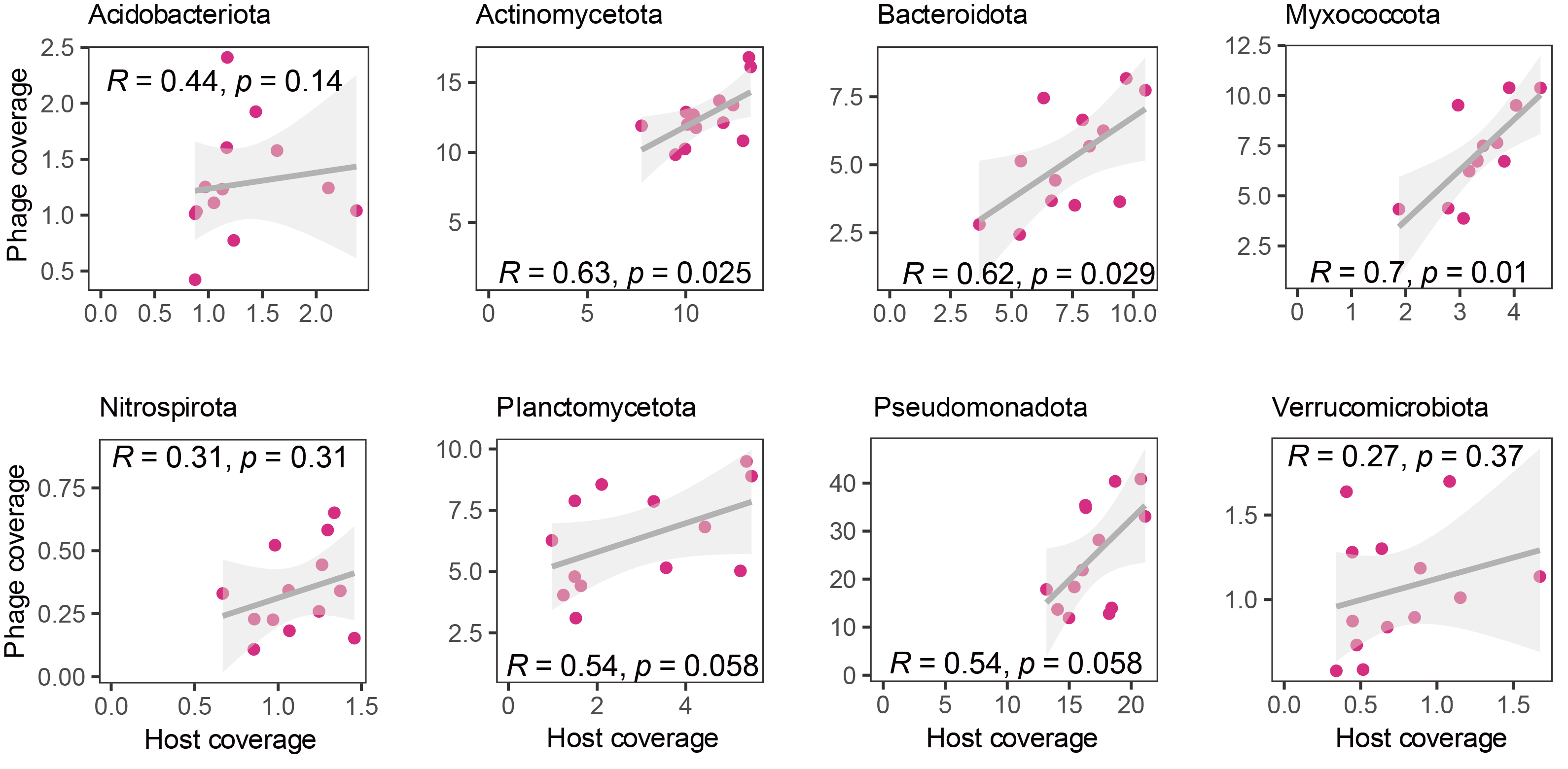


**Fig. S8** Spearman correlation analysis between host and phage coverage at the host phylum level


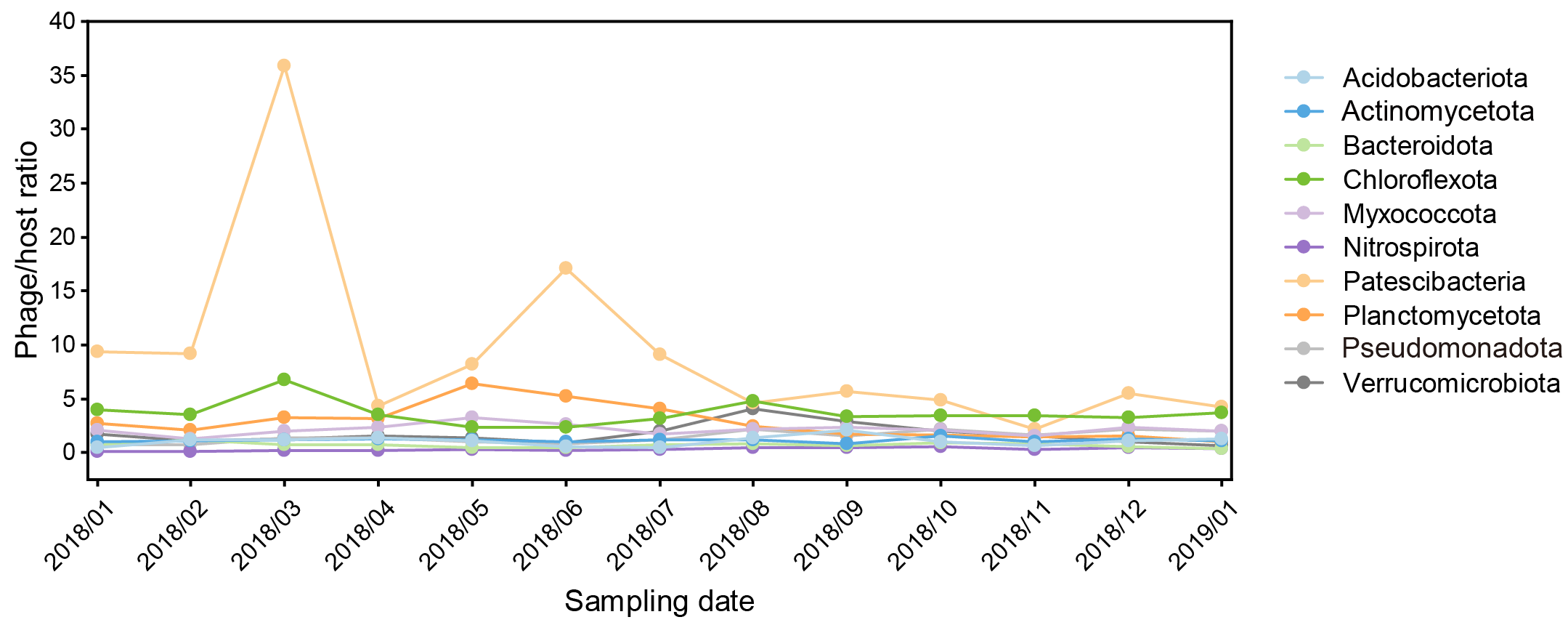


**Fig. S9** Variations in phage/host ratios over time at the phylum level


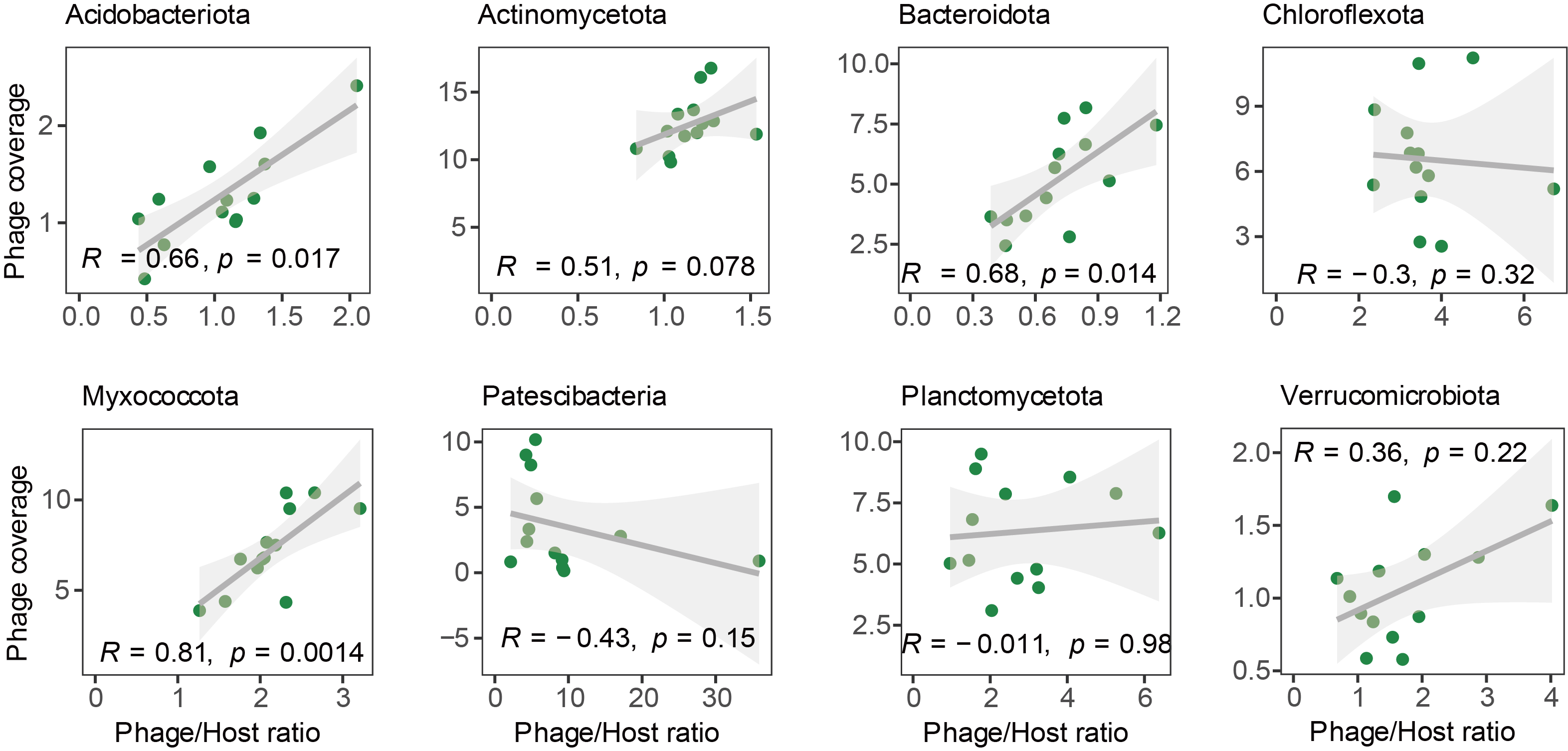


**Fig. S10** Spearman correlation analysis between phage/host ratio and phage coverage at the host phylum level


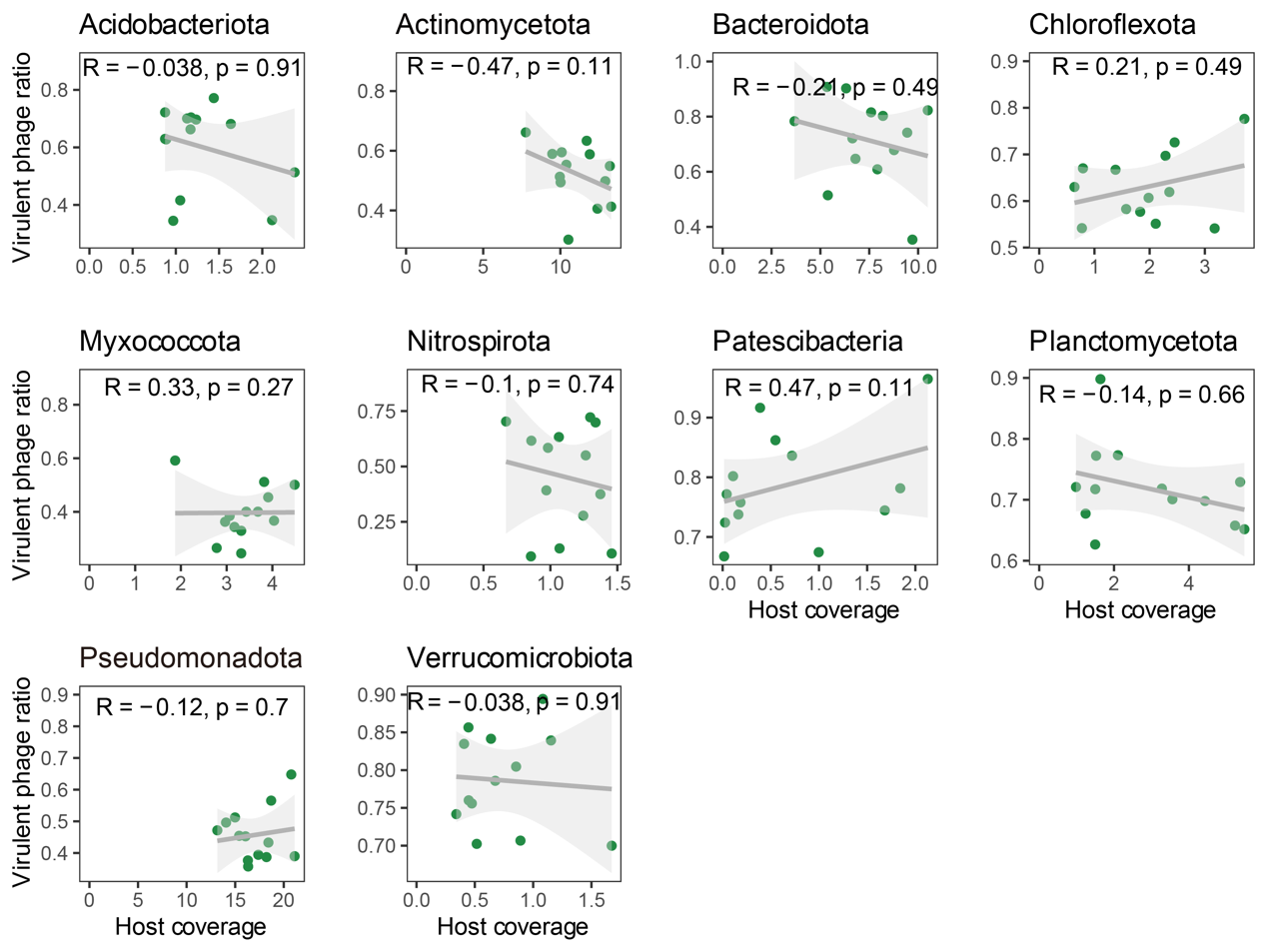


**Fig. S11** Spearman correlation analysis between host coverage and virulent phage ratio at the host phylum level

**References**

1. Chen Y, Wang Y, Paez-Espino D, Polz MF, Zhang T. Prokaryotic viruses impact functional microorganisms in nutrient removal and carbon cycle in wastewater treatment plants. Nat Commun. 2021;12:5398. <https://doi.org/10.1038/s41467-021-25678-1>.

2. Wang D, Wang Y, Liu L, Chen Y, Wang C, Xu X, et al. Niche differentiation and symbiotic association among ammonia/nitrite oxidizers in a full-scale rotating biological contactor. Water Res. 2022;225:119137. <https://doi.org/10.1016/j.watres.2022.119137>.

3. Nurk S, Meleshko D, Korobeynikov A, Pevzner PA. metaSPAdes: a new versatile metagenomic assembler. Genome Res. 2017;27:824-34. <https://doi.org/10.1101/gr.213959.116>.

4. Kolmogorov M, Bickhart DM, Behsaz B, Gurevich A, Rayko M, Shin SB, et al. metaFlye: scalable long-read metagenome assembly using repeat graphs. Nat Methods. 2020;17:1103-10. <https://doi.org/10.1038/s41592-020-00971-x>.

5. Walker BJ, Abeel T, Shea T, Priest M, Abouelliel A, Sakthikumar S, et al. Pilon: An Integrated Tool for Comprehensive Microbial Variant Detection and Genome Assembly Improvement. PLoS One. 2014;9:e112963. <http://doi.org/10.1371/journal.pone.0112963>.

6. Bertrand D, Shaw J, Kalathiyappan M, Ng AHQ, Kumar MS, Li C, et al. Hybrid metagenomic assembly enables high-resolution analysis of resistance determinants and mobile elements in human microbiomes. Nat Biotechnol. 2019;37:937-44. <https://doi.org/10.1038/s41587-019-0191-2>.

7. Lieberman-Aiden E, Van Berkum NL, Williams L, Imakaev M, Ragoczy T, Telling A, et al. Comprehensive mapping of long-range interactions reveals folding principles of the human genome. Science. 2009;326:289-93. <https://doi.org/10.1126/science.1181369>.

8. Press MO, Wiser AH, Kronenberg ZN, Langford KW, Shakya M, Lo C-C, et al. Hi-C deconvolution of a human gut microbiome yields high-quality draft genomes and reveals plasmid-genome interactions. biorxiv. 2017:198713. <https://doi.org/10.1101/198713>.

9. Li H, Durbin R. Fast and accurate long-read alignment with Burrows–Wheeler transform. Bioinformatics. 2010;26:589-95. <https://doi.org/10.1093/bioinformatics/btp698>.

10. Faust GG, Hall IM. SAMBLASTER: fast duplicate marking and structural variant read extraction. Bioinformatics. 2014;30:2503-5. <https://doi.org/10.1093/bioinformatics/btu314>.

11. Li H, Handsaker B, Wysoker A, Fennell T, Ruan J, Homer N, et al. The sequence alignment/map format and samtools. Bioinformatics. 2009;25:2078-9. <https://doi.org/10.1093/bioinformatics/btp352>.

12. Uritskiy G, Press M, Sun C, Huerta GD, Zayed AA, Wiser A, et al. Accurate viral genome reconstruction and host assignment with proximity-ligation sequencing. bioRxiv. 2021. <https://doi.org/10.1101/2021.06.14.448389>.
